# The Impact of Microbial Biotransformation of Catechin in Enhancing the Allelopathic Effects of *Rhododendron formosanum*

**DOI:** 10.1371/journal.pone.0085162

**Published:** 2013-12-31

**Authors:** Chao-Min Wang, Tsai-Chi Li, Yun-Lian Jhan, Jen-Hsien Weng, Chang-Hung Chou

**Affiliations:** 1 Research Center for Biodiversity, China Medical University, Taichung, Taiwan; 2 Graduate Institute of Ecology and Evolutionary Biology, China Medical University, Taichung, Taiwan; 3 Department of Life Sciences, National Cheng Kung University, Tainan, Taiwan; Nanjing Agricultural University, China

## Abstract

*Rhododendron formosanum* is distributed widely in the central mountains in Taiwan and the major allelopathic compound in the leaves has been identified as (-)-catechin, which is also a major allelochemical of an invasive spotted knapweed in North America. Soil microorganisms play key roles in ecosystems and influence various important processes, including allelopathy. However, no microorganism has been identified as an allelochemical mediator. This study focused on the role of microorganisms in the allelopathic effects of *R. formosanum*. The microorganism population in the rhizosphere of *R. formosanum* was investigated and genetic analysis revealed that the predominant genera of microorganisms in the rhizosphere of *R. formosanum* were *Pseudomonas*, *Herbaspirillum*, and *Burkholderia*. The dominant genera *Pseudomonas* utilized (-)-catechin as the carbon source and catalyzed the conversion of (-)-catechin into protocatechuic acid *in vitro*. The concentrations of allelochemicals in the soil were quantified by liquid chromatography-electrospray ionization/tandem mass spectrometry. The concentration of (-)-catechin in the soil increased significantly during the extreme rainfall in the summer season and suppressed total bacterial populations. Protocatechuic acid accumulation was observed while total bacterial populations increased abundantly in both laboratory and field studies. Allelopathic interactions were tested by evaluating the effects of different allelochemicals on the seed germination, radicle growth, and photosynthesis system II of lettuce. Protocatechuic acid exhibited higher phytotoxicity than (-)-catechin did and the effect of (-)-catechin on the inhibition of seed germination was enhanced by combining it with protocatechuic acid at a low concentration. This study revealed the significance of the allelopathic interactions between *R. formosanum* and microorganisms in the rhizosphere. These findings demonstrate that knowledge regarding the precise biotransformation process of (-)-catechin by microorganisms in the environment is necessary to increase our understanding of allelopathy.

## Introduction

The concept of plant-soil interactions has become widely recognized as a major driving force of community composition and ecosystem functioning in the past decade. The term ‘plant-soil feedback’ has been coined to name the multiple interactions between plants and soil organisms and has been adopted by many ecologists [1,2]. The plant-soil feedback as a driver of plant community especially in the context of plant succession and invasion has been recognized as an important issue among ecologist. Positive plant-soil feedback includes the soil nutrient availability enhanced by soil microorganisms involved in mineralization or promotion of plant nutrient uptake [2]. In contrast, negative feedback includes the accumulation of pathogens, parasites and even phytotoxic chemicals, resulting in plant succession and invasion [3-5]. The negative feedback of one plant to another plant through the release of chemical compounds into the environment is termed ‘allelopathy’ [6]. This phenomenon is an important factor in forest tree dominance in terrestrial ecosystems [7].

Over the past decades, research on a major invasive spotted knapweed species, *Centaurea maculosa*, in western North America has led to a very significant ecological discovery. Callaway and Aschehoug (2000) compared the competitive effect of invasive spotted knapweed from Eurasia and North America on bunchgrass that co-existed with spotted knapweed. The invasive spotted knapweed had stronger inhibitory effects on bunchgrass from North America than on the native Eurasian species. The allelochemical from the root of *C. maculosa* was isolated and identified as catechin [8,9]. Based on this discovery, the ‘novel weapon hypothesis’ of invasive plants was proposed. Some invasive species can produce significant allelopathic substances for which native species have not yet evolved resistance [10,11] and native plant species may also evolve to tolerate the effects of an exotic invader [12,13]. Studies have claimed that the successful invasion of *C. maculosa* in non-native North America resulted from the release of the allelopathic compound, catechin [14,15]. The allelopathic role of catechin has been queried because it appears in the soil at low concentrations [14,15] and the phytotoxic effects of catechin on plants only occurred at high concentrations [16-18]. Recently, researchers have proposed that catechin appears to transform rapidly into other chemical forms once injected into soil [16] and decreases total culturable count numbers from the bacterial community and inhibits the growth of some, but not all, of the soil bacterial population tested [19]. The allelopathic effects of catechin may, instead, be due to the effect of soil microorganisms. All these results suggest that the phytotoxic effects of catechin may be mediated through the microbes in the soil. However, to date, the interactions between catechin and soil microbes are still unclear. 

*Rhododendron formosanum* is an endemic species distributed widely in the central mountains in Taiwan, between 800 and 2000 m above sea level (asl.). This species exhibits a pure stand and understory species are rare [20,21]. The soil is acidic (pH 4.0) and nitrogen availability is limited [22]. Previously, the major compound extracted from the leaves of *R. formosanum* was identified as (-)-catechin and its allelopathic potential was confirmed by measuring the germination rate and radicle growth of *Lactuca sativa* and *Bidens pilosa* [20,21]. In nature, (+)-catechin is the most commonly found enantiomer present in fruits, wine, and vegetables as well as in many nonfood plants. The difference between (+)-catechin and (-)-catechin is due to structural stereoisomer. The two enantiomers have identical nuclear magnetic resonance (NMR) spectra and high-performance liquid chromatography (HPLC) retention times. The only difference is in optical rotation, where these two compounds rotate plane polarized light equally in opposite directions. Previous studies indicated that (-)-catechin is a potent antioxidant and is more phytotoxic than (+)-catechin [8,23]. The mirror image differences in molecular shape of catechin can result in differences in biological activity [24]. Although the allelopathic potential of *R. formosanum* had been confirmed [20], the role of (-)-catechin in the allelopathic phenomenon of *R. formosanum* is not fully understood.

Based on previous studies, we hypothesized that decomposing microorganisms could be involved and play a significant role in the allelopathic effects of catechin. Therefore, the diversity of microorganisms associated with *R*. *formosanum* grown in a nutrient-limited ecosystem was explored. A combination of culture-dependent and culture-independent methods was used to determine bacterial diversity and to analyse their function in the rhizosphere of *R*. *formosanum*. Furthermore, the process behind (-)-catechin biotransformation and the metabolites produced by the dominant bacteria were investigated. This study, therefore, mainly concentrated on the effects of the (-)-catechin biodegradation process on ecological interactions, with particular emphasis on the role of microorganisms in the rhizosphere of *R*. *formosanum*.

## Materials and Methods

### Sample collection

The litter and organic matter layers of *R. formosanum* were collected in December 2009, August 2010, December 2010, August 2011, December 2011, July 2012, and August 2012 at the Sunlinksea site (23°38'12.40"N, 120°47'46.50"E at 1775 m asl.; annual rainfall: 2000–2300 mm) and the Dasyueshan site (24°14'6.49"N, 120°57'7.29"E at1911 m asl.; annual rainfall: 2000–3000 mm). A 100 g soil sample was stored at 4°C for microbial community analysis and a 1 kg soil sample was air-dried for chemical analysis. The precipitation of rainfall per month was obtained from the databank of the Central Weather Bureau of Taiwan. No specific permissions were required for these locations/activities. Those sampling sites are not in the region of National parks or any protected areas that required permissions to collect samples. In addition, *R. formosanum* is an endemic species, but not endangered or protected species in Taiwan. The litter and organic matter layers we collected that did not affect the growth of *R. formosanum*. No protected species were sampled in this study.

### Microbial community analysis in the rhizosphere of *R. formosanum*

The rhizosphere microbial DNA was extracted (1 g soil from each sample) using a ZR Soil Microbe DNA MiniPrep kit (Zymo Research, Irvine, CA) and following the manufacturer’s instructions. The DNA from all samples were pooled together and further subjected to polymerase chain reaction (PCR) amplification using the 16S ribosomal RNA gene of bacteria with the 27F and 1492R primer pair combinations [25]. Each PCR reaction mixture contained 1.5 mM MgCl_2_, 0.2 mM dNTP, 10 mM Tris-HCl (pH 9.0), 50 mM KCl, 100 ng of DNA template, 20 pM of primer and 2 U of *Taq* DNA polymerase (Promega, Madison, WI) in a final volume of 50 µL. After 30 s denaturation at 94°C, the reaction mixture was subjected to 30 cycles of 50°C for 1.5 min, 72°C for 1.5 min, and 94°C for 1.5 min, followed by a final extension at 72°C for 5 min using a Perkin Elmer GeneAmp 9600 PCR system (Applied Biosystems, Foster City, CA). 

The PCR products were purified with a QIAquick PCR purification kit (Qiagen, Valencia, CA) and cloned into the pCR2.1 vector using a TOPO TA cloning kit (Invitrogen, Carlsbad, CA). Plasmids were transformed into the *Escherichia coli* strain TOP10’ (Invitrogen) and 16S rDNA gene fragments were re-amplified using the M13 forward and reverse primer pair. The amplified fragments (1056 clones in total) were further digested by *Hae*III and *Eco*RI for restriction fragment length polymorphism (RFLP) analysis. The clones containing differential DNA fragments were sequenced using an automated sequencer (ABI 3730, Applied Biosystems). All the sequences were submitted to GenBank via Sequin software. Strains of uncultured bacteria identified by direct DNA extraction from 3 sampling sites of *R. formosanum* were assigned RF3. The GenBank accession numbers for the sequences of uncultured clones identified in this study are JN 379390 to JN 379430 for the 16S rRNA gene. 

### Enrichment and isolation of catechin-degrading microorganisms

Catechin-degrading microorganisms were isolated using modified W medium [26] with 0.1% catechin as the only carbon source. A mixture of 10 g soil and 90 mL modified W medium was incubated at 30°C and shaken on a shaker at 150 rpm for 7 d. After incubation, 10 mL of the original mixture was further inoculated into 90 mL of W medium, containing 0.1% catechin, and incubated at 30°C for another 7 d. The inoculation and incubation process was repeated for 3 months until the catechin in the W medium could not be detected by thin-layer chromatography. 

### Identification and characterization of catechin-degrading microorganisms

The catechin-degrading microorganisms were cultured on trypticase soy agar (TSA) for DNA extraction. Chromosomal DNA was extracted from bacterial isolates and further subjected to PCR amplification using the 16S ribosomal RNA gene with the 27F and 1492R primer pair combinations as described previously [25]. The PCR products were purified and cloned into the pCR2.1 vector using the TOPO TA cloning kit (Invitrogen). Plasmids were transformed into the *Escherichia coli* strain TOP10’ (Invitrogen) and the 16S rDNA gene fragments were re-amplified using the M13 forward and reverse primer pair. The clones containing differential DNA fragments were sequenced using an automated sequencer (ABI 3730, Applied Biosystems). Nucleotide sequences of the 16S rRNA gene were aligned using Clustal W and phylogenetic trees were constructed using MEGA 5.0 [27] software with a neighbour-joining algorithm and 1000 bootstrap analysis. The sequences were further examined using Pintail 1.0 software [28] in order to detect chimeras and other anomalies. Strains of catechin-utilizing bacteria isolated from 3 sampling sites of *R. formosanum* were assigned as CRF3. The accession numbers in GenBank for the cultured strains are JQ010849 to JQ010855.

### Biotransformation of catechin

*Pseudomonas* sp. CRF3-Ps-1 (5×10^5^ CFU/mL) was inoculated into W medium (200 mL), containing 0.1% catechin, and was grown aerobically at 30°C for 72 h in a shaking (150 rpm) incubator. After centrifugation at 10 000 g for 10 min, the supernatant was filtered through a 0.22 µm membrane (Millipore, Billerica, MA) and the proteins precipitated using acetone. The supernatant was partitioned with ethyl acetate and concentrated, yielding 247.97 mg crude residue. The residue was then passed through a 2.5 cm × 92 cm MCI gel CHP20P column (Supelco Inc. Bellefonte, PA) using a 1-step gradient of H_2_O in methanol. Further purification was accomplished using silica gel 60 (Merck, Darmstadt, Germany) and a RP-18 (Merck) column to obtain purified compounds that were structurally elucidated by 400 MHz nuclear magnetic resonance (NMR) spectroscopy (Bruker Avance III) and electrospray ionization tandem mass spectrometry (ESI-MS/MS) analysis (Esquire HCT, Bruker Daltonics, Billerica, MA). 

### Quantification of allelochemicals

Ten grams of *Rhododendron formosanum* soil was extracted by vortexing in 90 mL methanol at 150 rpm for 24 h. After centrifugation for 10 min at 4500 rpm, the supernatant was passed through 0.22 µm filters (Millipore) and placed in sample vials for liquid chromatography (LC) analysis. LC mass analysis for the quantification of catechin and protocatechuic acid in soil was carried out with an Atlantis T3 RP-18 column (150 × 2.1 mm; 3µm; Waters, Milford, MA). The column was eluted with buffer A (distilled water/acetonitrile/formic acid; 95/5/0.1, v/v/v) and buffer B (acetonitrile/formic acid; 100/0.1, v/v) at a flow rate of 0.25 mL/min at 25°C. The column was eluted initially with 100% buffer A, followed by a linear increase in buffer B to 10% from 0 to 5 min, and maintained in 10% buffer B for another 5 min. From 10 to 20 min, a linear increase in buffer B to 20% was carried out and the column was maintained in 20% buffer B for another 10 min. The column was further eluted with a linear increase in buffer B to 50% from 30 to 35 min and maintained for another 5 min. From 40 to 45 min, buffer B was increased linearly from 50% to 95% and maintained for 5 min. The column was finally equilibrated with buffer A for 10 min. Quantification of the metabolites in soil was performed in the ion monitoring mode selected. Both positive and negative ionization mode MS analyses were undertaken. The temperature of the ion source was maintained at 100°C. The dry temperature was 365°C and the desolvation gas, N_2_, had a flow rate of 12 L/min. Product ion scans for mass were performed by low-energy collision (20 eV) using argon as the collision gas. All liquid chromatography-electrospray ionization/tandem mass spectrometry (LC-ESI-MS/MS) data were processed by Bruker Daltonics Data analysis software (version 4.0). The molecular ion peaks and mass spectra recorded were compared to those of reference substances. All chemicals were prepared at a concentration range of 1–1000 µg/mL. From 2009 to 2012, the concentration of (-)-catechin in the soil of *R. formosanum* was measured using LC-ESI-MS/MS analysis. The linearity of the calibration curves was demonstrated by the good determination of coefficients (*r* ^2^) obtained for the regression line. Good linearity was achieved over the calibration range, with all coefficients of correlation greater than 0.995. All samples were freshly prepared. The mean values for the regression equation for (-)-catechin were y = 26464x + 25580 (*r* ^2^ = 0.9992). The mean values for the regression equation for protocatechuic acid were y = 5786.7x + 4002.6 (*r* ^2^ = 0.9985).

### Recovery and half-life determination of catechin and protocatechuic acid

The fresh soil from the rhizosphere of *R. formosanum* was prepared for recovery and half-life of catechin and protocatechuic acid determination. Standard (-)-catechin or protocatechuic acid was added in soil at known concentrations (200 and 25 µg/g soil). Soil with catechin or protocatechuic acid was further extracted by methanol. All samples were filtered through 0.22 µm Millipore membrane filter for further analysis. A 5.0 µL aliquot of the sample solution was analysed by LC/MS and three replicates were completed. The recovery % of standard (-)-catechin or protocatechuic acid was determined as: [(measured mass concentration-basal (-)-catechin or protocatechuic acid mass concentration)/added (-)-catechin or protocatechuic acid mass concentration]×100%. For half-life determination of chemicals, fresh soil (10 g) with standard (-)-catechin or protocatechuic acid were incubated for 120 hours. Soil was extracted by methanol and the concentration of catechin and protocatechuic acid was measured at 1, 3, 5, 7, 24, 48, and 120 hours after incubation.

### Bioassay of phytotoxicity

The allelopathic effects of the separated compounds were assayed by the standard sponge bioassay [29]. Seeds of *Lactuca sativa* were used and seedling radicle growth inhibition was measured after 72 h incubation. Each test contained 10 seeds and the bioassay was replicated 3 times for each measurement. The toxicity of each compound (effective concentration 50, EC_50_) was estimated using a dose-response curve with at least 8 concentrations spanning the range from no response to total inhibition of root growth. The synergistic effect of (-)-catechin with protocatechuic acid was tested with different binary mixtures according to the concentration of allelochemicals in the soil. 

### Chlorophyll fluorescence measurements

Cucumber (*Cucumis sativus*) seeds were grown in a growth chamber at 25°C, with a 12/12 h (light/dark) photoperiod, and 150 µmol photon /m^2^ s irradiance for 10 days. Ten-day-old seedlings were maintained in solutions containing different concentrations of catechin and protocatechuic acid. The inhibitory effect of catechin and protocatechuic acid on photosynthetic capacity was evaluated by analysing chlorophyll fluorescence parameters. All fluorescence measurements were performed using the portable chlorophyll fluorescence analyser PAM-2100 (Pulse Amplitude Modulate fluorometer; Walz Heinz GmbH, Effeltrich, Germany). The instrument, which contains a built-in light source, provides information to produce rapid light curves (RLCs). Experimental data were collected in a sequence of actinic illumination with light intensities increasing from 5 to 2,300 µmol/m^2^s in 10 steps, and each illumination period lasted 10 s. F_v_ / F_m_ (maximum quantum efficiency of PSII), F_0_ (initial fluorescence), F_m_ (maximum fluorescence), and ETR (electron transport rate) are reliable indicators of plant health [30,31]. The following formula was used to calculate the chlorophyll parameters [30,31]: 

F_v_/F_m_ = (F_m_ - F_0_)/F_m_

ETR = quantum yield × 0.84 × 0.5 × PPFD (photosynthetic photon flux density). PFD is the photon flux density of photosynthetically active radiation, 0.84 is the assumed light absorbance of the sample, and 0.5 corrects for 2 quanta of light required for the transport of 1 electron.

### Data analysis

All statistical analyses were conducted using SPSS 13.0. All results have been expressed as average mean ± standard error (S.E.) values. Analysis of variance (ANOVA) was used to evaluate differences, and a P value of <0.05 was considered statistically significant. Duncan’s multiple range test was also used to evaluate the means; the same letters represent values that were not significantly different at the α = 0.05 level. Pearson correlation was used to calculate the relationship coefficients.

## Results

### The correlation between the concentration of (-)-catechin, monthly precipitation and bacterial population in the soil around *R. formosanum*

**Figure 1:**
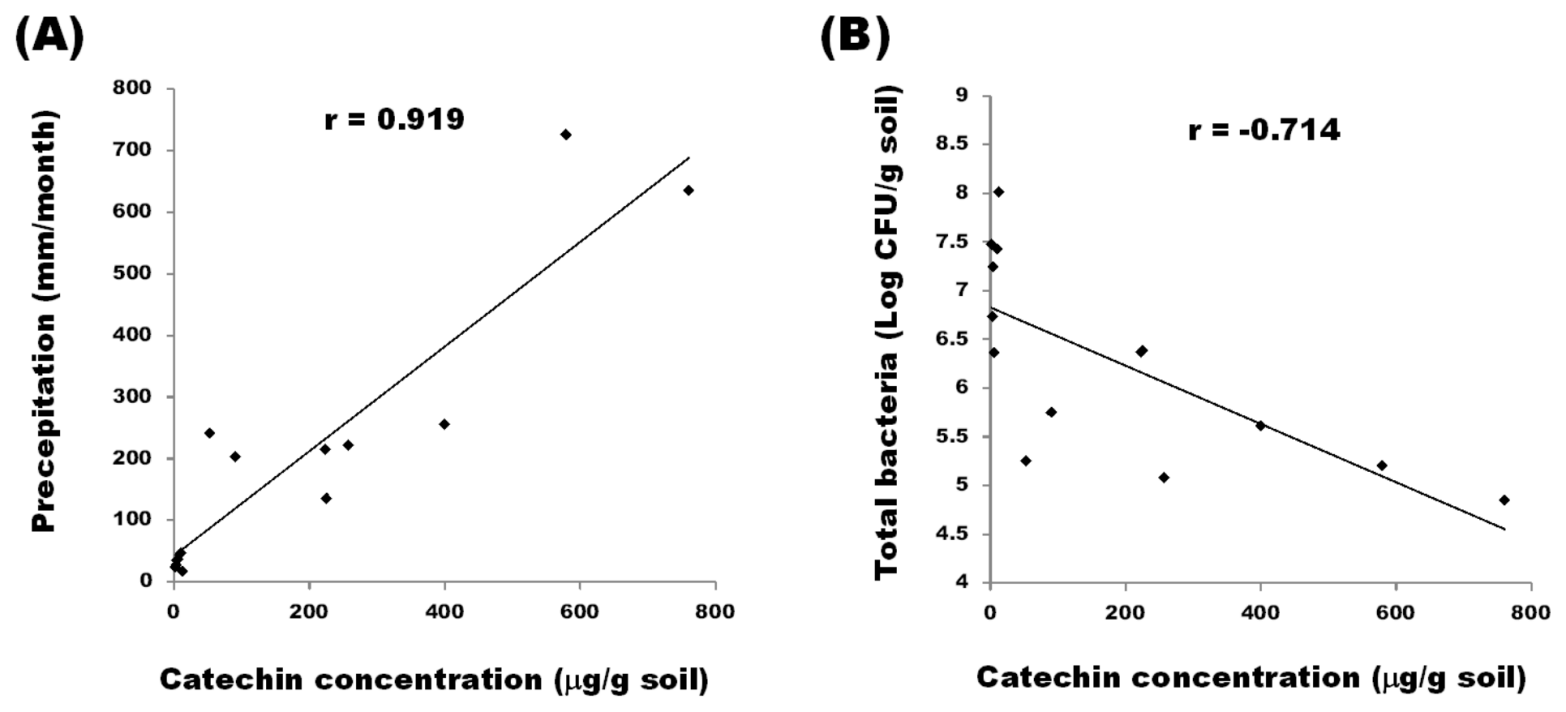
Pairwise correlations between (-)-catechin concentration, monthly precipitation, and bacterial populations. (A) Correlation between the concentration of (-)-catechin in soil of *R. formosanum* and monthly precipitation (*r* = 0.919, *P* < 0.0001, *n* = 14). (B) Correlation between the concentration of (-)-catechin and bacterial populations in soil of *R. formosanum* (*r* = -0.714, *P* = 0.0041, *n* = 14).

From 2009 to 2012, the concentration of (-)-catechin in the *R. formosanum* soil was measured by LC-MS/MS analysis. The linearity of calibration curves was demonstrated by the good determination of coefficients (*r* ^2^) obtained for the regression line. Correlations were also calculated between the concentration of (-)-catechin, monthly precipitation, and the number of colony forming units (CFUs) of soil bacteria. As shown in Figure 1, the concentration of catechin was significantly correlated to precipitation at the studied sites (*r* = 0.919, *P* < 0.0001, *n* = 14) (Figure 1A.). This result indicates that the concentration of (-)-catechin increased with monthly precipitation. The amount of soil bacteria (log CFU/g soil) was negatively correlated with catechin concentration in the studied sites (*r* = -0.714, *P* = 0.0041, *n* = 14) (Figure 1B).

### Bacterial flora in the *R. formosanum* rhizosphere

**Figure 2:**
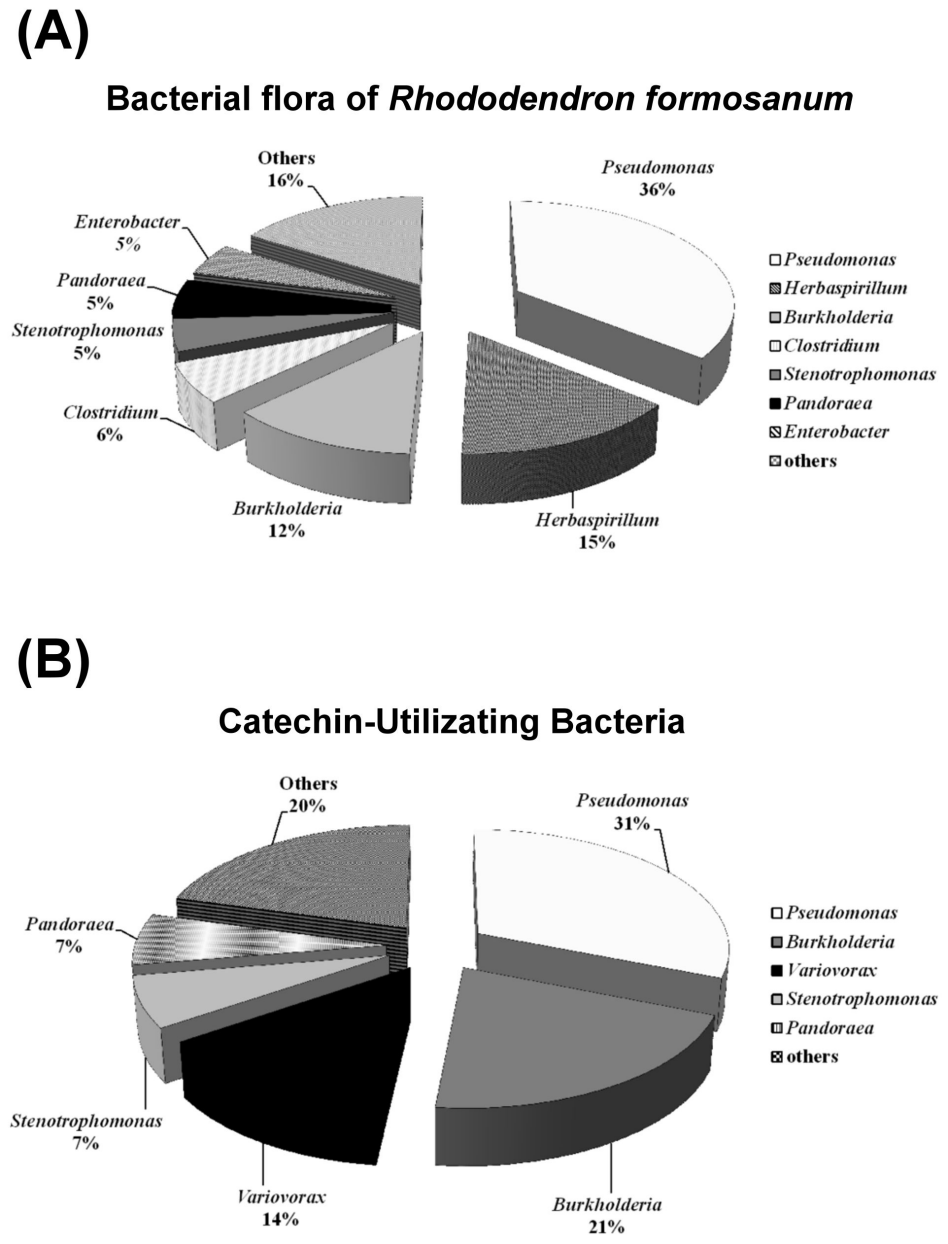
Bacterial flora (A) and catechin utilizing bacteria (B) in the rhizosphere of a *Rhododendron formosanum* plantation. (-)-Catechin was the only carbon source added to the medium that was used for microbial isolation. After 3 months of incubation, *Pseudomonas* spp., *Burkholderia* spp., *Variovorax* spp. *Stenotrophomonas* spp., and *Pandoraea* spp. were isolated and identified as dominant catechin-utilizing bacteria.

The bacterial flora was analysed in order to understand the interactions between allelopathic compounds and microorganisms in the rhizosphere of *R. formosanum*. The soil around *R. formosanum* plants was collected from 3 different sites and the bacterial flora analysed using 16S rRNA gene sequencing. As shown in Figure 2A, the relative abundance of bacterial genera in the soil of *R. formosanum* was expressed as relative percentages. The predominant genera were *Pseudomonas* (36%), *Herbaspirillum* (15%), *Burkholderia* (12%), *Clostridium* (6%), *Stenotrophomonas* (5%), *Pandoraea* (5%) and *Enterobacter* (5%). Other genera (16%) were also present, including *Achromobacter*, *Alcaligenes*, *Desulfosporosinus*, *Duganella, Dyella*, *Sporotalea*, *Steroidobacter* and *Variovorax*. Only 10% of the total bacteria in the rhizosphere were gram-positive bacteria. In conclusion, the most dominant bacteria in the rhizosphere of *R. formosanum* were gram-negative proteobacteria, especially *Pseudomonas* spp. 

### (-)-Catechin-utilizing bacteria

(-)-Catechin was the only carbon source added to the medium for microbial isolation. After 3 months of incubation, *Pseudomonas* spp., *Burkholderia* spp. and *Variovorax* spp. were isolated and identified as dominant catechin-utilizing bacteria (Figure 2B). The catechin-utilizing bacteria were assigned to CRF3 and found to be β- and γ-proteobacteria (Figure S1). In the β-proteobacteria cluster, families of *Oxalobacteraceae*, *Comamonadaceae*, *Burkholderiaceae* and *Alcaligenaceae* were often found but the catechin-utilizing bacteria were restricted to the *Comamonadaceae* and *Burkholderiaceae* families. In the *Comamonadaceae* family, catechin-utilizing bacterium, *Variovorax* CRF3-Va-1, was related to *Variovorax boronicumulans* and the uncultured clone RF3-C1 (Figure S1). In the γ-proteobacteria cluster, the family *Pseudomonadaceae* was abundant in the rhizosphere of *R. formosanum*. The strains of catechin-utilizing bacteria were aligned closely with the uncultured clones, RF3-C2, RF3-C4, and the type strain, *Pseudomonas asplenii* (Figure S2). 

### Biotransformation process of (-)-catechin

In order to understand the biotransformation of (-)-catechin, the dominant *Pseudomonas* sp. CRF3-Ps-1 was selected as a model bacterium to investigate the effects of allelochemicals, including biotransformation intermediates and bacterial metabolites. Several catechin biotransformation intermediates were isolated and identified by NMR spectroscopy and LC-ESI-MS/MS (Text S1; Tables S1-S4; Figures S3-S6). On LC-ESI-MS/MS fragment analysis (Tables S1-S4; Figures S3-S6), the spectra generated by the compounds in this study gave the deprotonated molecule [M-H]^-^, the protonated molecule [M+H], and the sodium adduct [M+Na]. In tandem mass spectrometric mode (Table S5), catechin produced the deprotonated form [M-H]^-^ (*m/z* 289) and lost a CH_2_CHOH (*m/z* 245) group [32]. Taxifolin contained the deprotonated ion (*m/z* 303), and fragments corresponding to deprotonated luteolin (*m/z* 285), 5,7-dihydroxychromone (*m/z* 177), and 1,3,5-trihydroxybenzene (*m/z* 125) [33]. CO_2_ loss was observed in protocatechuic acid and, as a result, the characteristic [M-H-44]^-^ (*m/z* 109) ion was formed [32].. ESI ionization was more sensitive in the positive mode than in the negative mode for glycerol ionization. For example, a [M+Na] (*m/z* 115) ion was generated as a characteristic ion for glycerol in the positive mode (Table S5). 

**Figure 3:**
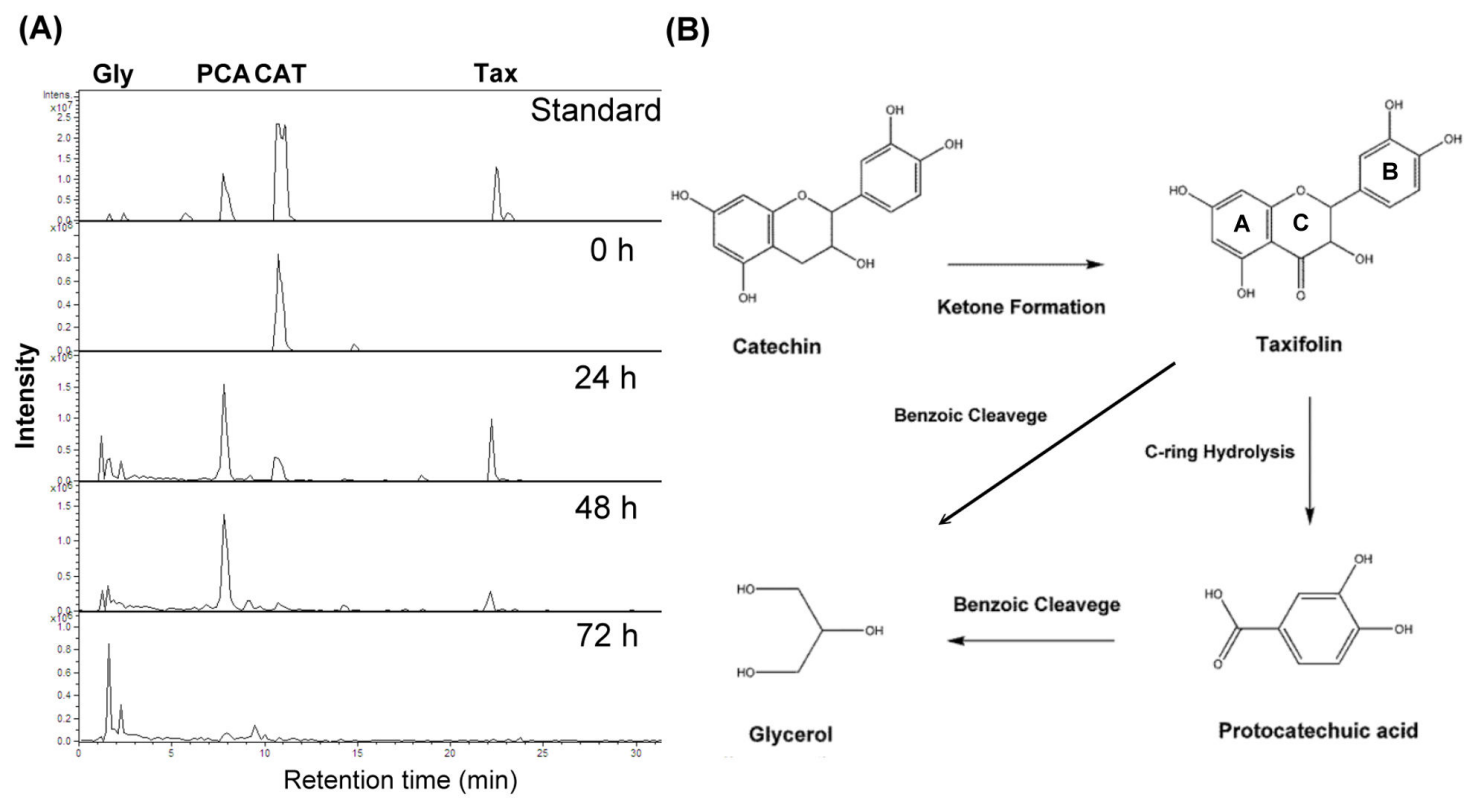
Metabolic pathway for (-)-catechin transformed by *Pseudomonas* sp. CRF3-Ps-1 was analysed by the LC-ESI-MS/MS method (A). (-)-Catechin (CAT) was transformed into taxifolin (Tax) via ketone formation during the first 24 h. Subsequently, C-ring hydrolysis occurred and generated protocatechuic acid (PCA) and glycerol (Gly). Finally, (-)-catechin was transformed into glycerol 72 h after incubation. The possible transformation hypothesis is also illustrated (B).

In conclusion, catechin was initially degraded into taxifolin via ketone formation at the C-ring. Later on, C-ring hydrolysis converted taxifolin into protocatechuic acid and glycerol. Finally, glycerol, the end product, was also generated by benzoic cleavage of protocatechuic acid (Figure 3). 

### Concentrations of allelochemicals *in vitro* and *in vivo*

**Figure 4:**
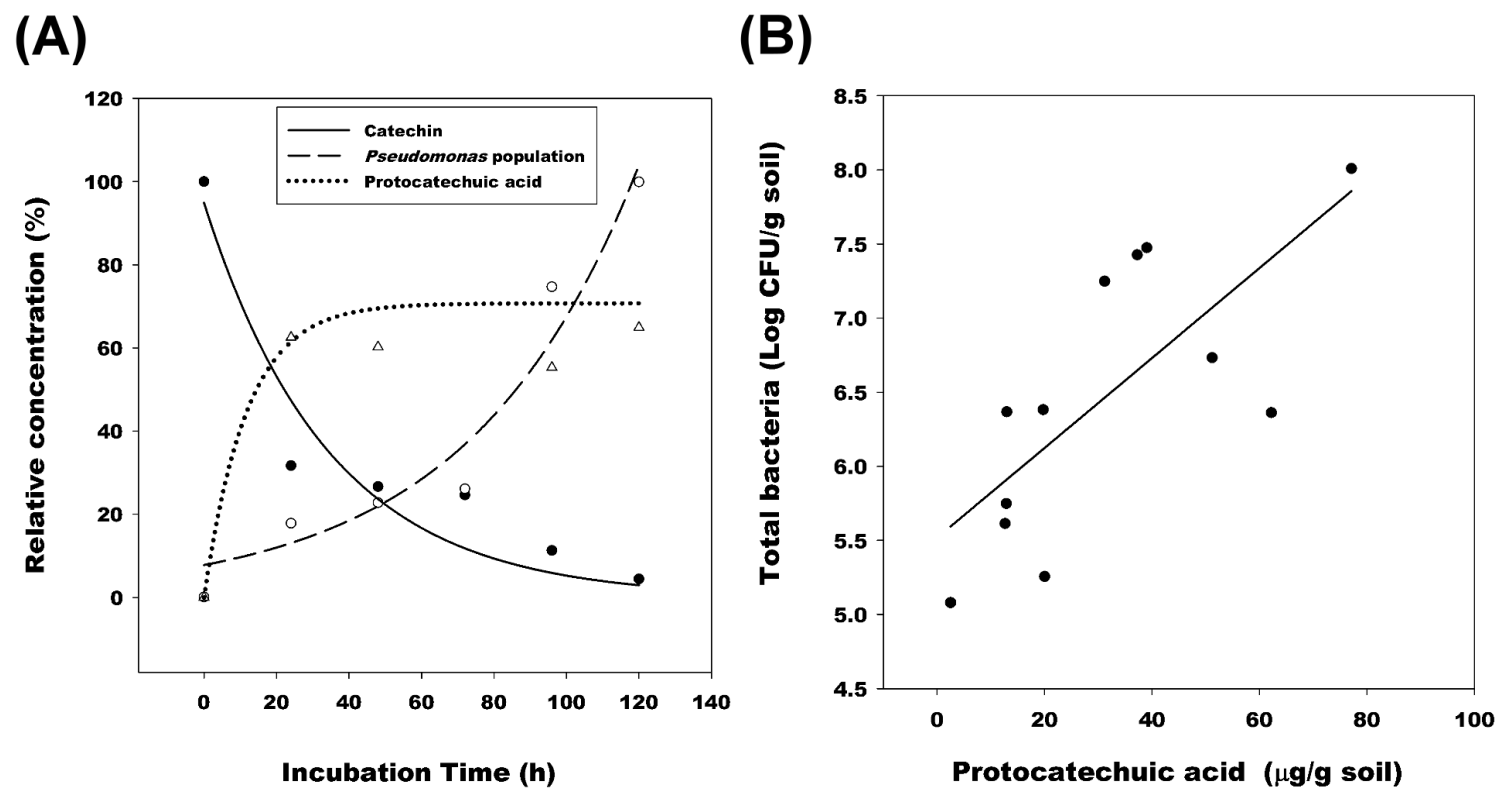
Relative concentrations of (-)-catechin and protocatechuic acid, and the bacterial population in the medium, during 120 h incubation with *Pseudomonas* sp. CRF3-Ps-1. (●) Relative concentration of catechin (*r* = -0.958, *P* = 0.0025, *n* = 6); (○) Bacterial population of *Pseudomonas* CRF3-Ps-1 (*r* = 0.974, *P* = 0.001, *n* = 6); (△) Relative concentration of protocatechuic acid (*r* = 0.874, *P* = 0.0226, *n* = 6). (B) Correlations between the concentration of protocatechuic acid and bacterial populations in the soil of *R. formosanum* (*r* = 0.734, *P* = 0.0066, *n* = 12).

 The biotransformation of catechin by *Pseudomonas* sp. CRF3-Ps-1 was studied. HPLC and LC-mass analysis were conducted to quantify the metabolites in the medium with the selected ion monitoring mode. Both positive and negative ionization mode MS analyses were undertaken. As shown in Figure 4A, the relative concentration of (-)-catechin in the medium was decreased immediately to 31.6%, while the concentration of protocatechuic acid was increased to 60 %, 24 h after inoculation. The bacterial population also increased slightly within 120 hours. In the field, the soil bacterial population positively correlated with protocatechuic acid concentration (*r* = 0.734, *P* = 0.0066, *n* = 12) (Figure 4B). These results indicate that the bacteria in the rhizosphere could transform (-)-catechin and accumulate protocatechuic acid in the soil. 

 As shown in Table 1, the concentration of catechin in the summer season was 178.36 ± 63.37 µg/g soil in Sunlinksea and 237.44 ± 89.49 µg/g soil in Dasyueshan; in winter, the concentration of catechin was as low as 5.30 ± 2.68 and 6.94 ± 2.95 µg/g soil, respectively. The concentration of protocatechuic acid was 35.79 ± 2.37 and 63.5 ± 7.5 µg/g soil, respectively, in winter and 14.07 ± 5.78 and 12.82 ± 0.09 µg/g soil, respectively, in summer season for these regions. Catechin in both sites was relatively lower in the summer season while the concentration of protocatechuic acid was higher during the winter season. Catechin can leach into soil during extreme rainfall, and the highest concentration of catechin was observed in the typhoon season (Table 1). Moreover, protocatechuic acid accumulated in the winter season but flowed away with the rain during the summer season (Table 1).

**Table 1:** The concentration of allelochemicals in the soil of *Rhododendron formosanum* in Sunlinksea and Dasyueshan.

Study Site	Season	Catechin (µg/g soil)	Protocatechuic acid (µg/g soil)	Precipitation (mm/month)
Sunlinksea				
	Winter	5.30 ± 2.6	35.79 ± 2.3	35.67 ± 6.5
	Summer	178.36 ± 63.3*	14.07 ± 5.7*	199.77 ± 32.3**
	Typhoon	760	<1	635
Dasyueshan				
	Winter	6.94 ± 2.9	63.5 ± 7.5	27.37 ± 5.6
	Summer	237.44 ± 89.5	12.82 ± 0.1	224.93 ± 16.0***
	Typhoon	579	<1	726

Results are the mean ± SE values of 3 experiments. Asterisks indicate significant difference (*p < 0.05, **p < 0.01, ***p < 0.005) between seasons.

### Recovery and half-life determination of catechin and protocatechuic acid

As shown in Table 2, the recovery values of (-)-catechin or protocatechuic acid were determined by adding 200 and 25 µg/g soil of each standard (-)-catechin or protocatechuic acid into two parts of the fresh soil. The average of the recovery of (-)-catechin and protocatechuic acid was 90.1% and 91.5%, respectively. In addition, catechin was degraded rapidly in the rhizosphere of *R. formosanum* (Figure S8) and the half-life of (-)-catechin in soil was 4.8 hours. Protocatechuic acid was more resistant to microbial degradation and the half-life of protocatechuic acid in soil was over 120 hours.

**Table 2:** The recovery value and half-life of standard (-)-catechin or protocatechuic acid in the soil from rhizosphere of *R. formosanum*.

Compound	(-)-Catechin	Protocatechuic acid
Added concentration (µg/g soil)	200	25
Measured mass concentration (µg/g soil)	180.2 ± 10.2	22.9 ± 1.1
Recovery (%)	90.1	91.5
RSD^*^ (%)	5.0	4.4
Half-life in soil (hr)	4.8 ± 1.2	>120

* RSD: relative standard deviation

### Phytotoxic effects of (-)-catechin and protocatechuic acid

**Figure 5:**
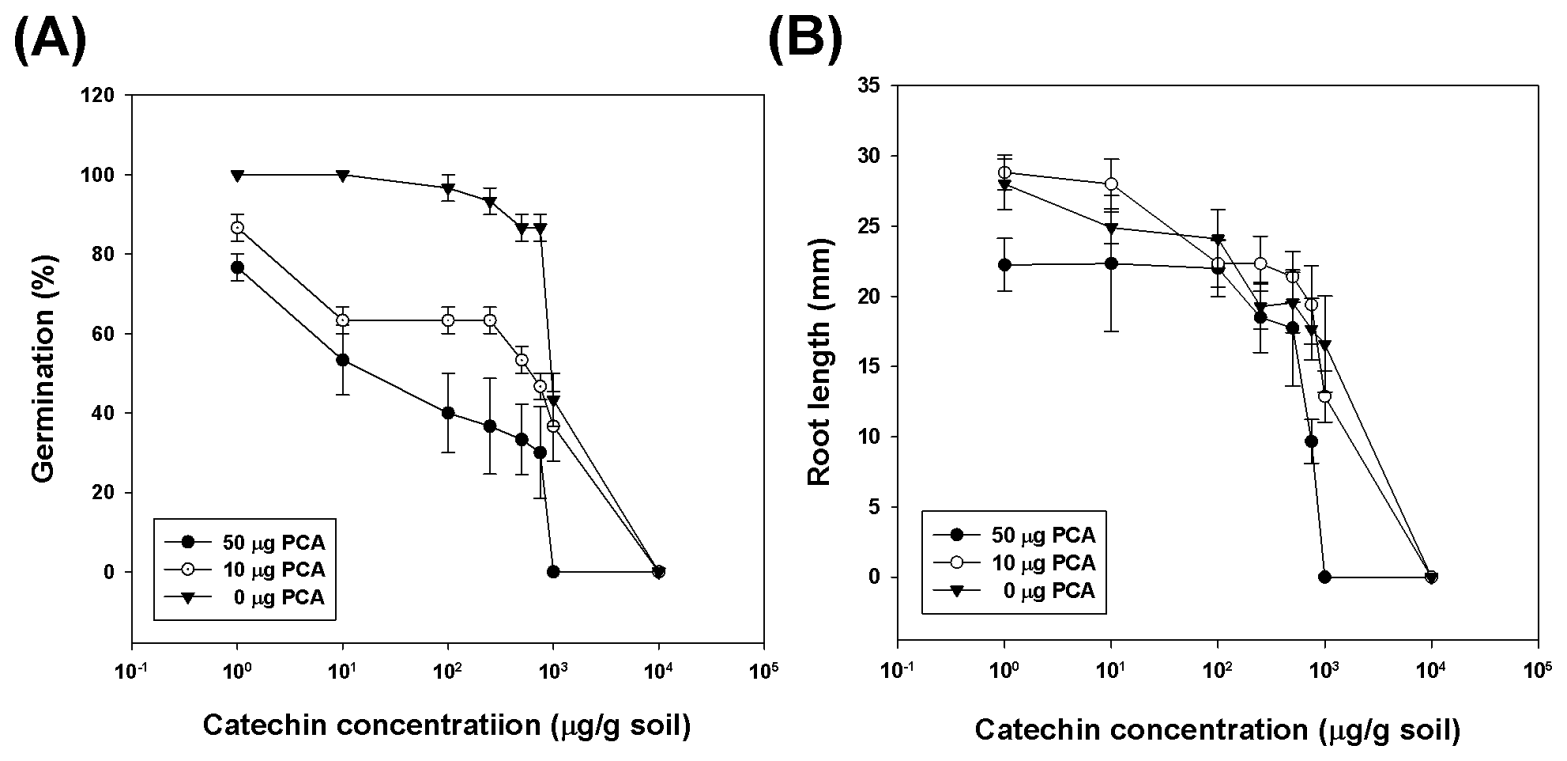
The phytotoxic effects of (-)-catechin on the seed germination (A) and radicle growth (B) of *Lactuca sativa* at different concentrations in combination with 0 µg, 10 µg and 50 µg protocatechuic acid. Error bars represent the standard errors of the mean.

Inhibitory effects of (-)-catechin and protocatechuic acid on radicle length, germination, and electron transport rate (ETR_max_) of *L. sativa* were compared by EC_50_ calculation. As shown in Table 3, protocatechuic acid had a significant phytotoxic effect on radicle growth and germination rate in *L. sativa* than did (-)-catechin (*P* < 0.05). The effects of (-)-catechin and protocatechuic acid on photosynthetic capacity were determined by analysing chlorophyll fluorescence parameters after 1 week of treatment with 4 concentrations of (-)-catechin and protocatechuic acid. Healthy plants typically achieve a maximum F_v_/F_m_ value of 0.84, and values lower than that are considered to indicate reduced capacity for the photochemical quenching of energy within PSII [31]. There were no significant changes in F_m_, but a significant reduction in ETR_max_ was observed in response to both (-)-catechin and protocatechuic acid treatments (Figure S7). In conclusion, protocatechuic acid inhibited the PSII system by decreasing electron transfer activity. In addition, a synergistic effect of (-)-catechin and protocatechuic acid was also observed. Figure 5A, B illustrates dose-response curves for 3 different concentrations of protocatechuic acid with (-)-catechin on germination rate and radicle length. The dose-response curves demonstrated that 50 µg/g soil of protocatechuic acid combined with 10–1000 µg/g soil of (-)-catechin caused over 46.7% inhibition of seed germination of *L. sativa*. Radicle length inhibition (Figure 5B), however, was not found to have any significant synergistic effects when both allelochemicals were combined. Furthermore, in order to understand the realistic effects of allelochemicals in soil, the inhibitory effects of (-)-catechin with protocatechuic acid were conducted with different binary mixtures according to the concentration of allelochemicals in the soil (Figure 5, Table S6). Treatments 2 and 3 were conducted to imitate the conditions in summer and winter. In the summer, 10 µg/g soil of protocatechuic acid combined with 250 and 750 µg/g soil of (-)-catechin caused 36.7% and 53.3% inhibition of seed germination, respectively. In the presence of 50 µg/g soil of protocatechuic acid during winter, 10 and 250 µg/g soil of (-)-catechin caused 46.7% and 63.4 % inhibition, respectively. In conclusion, biologically significant inhibition on seed germination was observed when both allelochemicals were combined.

**Table 3:** Inhibitory effects of (-)-catechin and protocatechuic acid on the radicle length, germination, and ETR_max_ of *L. sativa* at the EC_50_ concentration.

		EC_50_ (mM)	
	Radicle	Germination	ETR_max_
(-)-Catechin	10.8 ± 1.8	9.2 ± 1.3	> 20
Protocatechuic acid	4.4 ± 0.3*	4.3 ± 0.4*	3.2 ± 0.03

Results are the mean ± SE values of 3 experiments. Asterisk indicates significant difference (p < 0.05) between treatments.

## Discussion

### The role of (-)-catechin in the soil of *R. formosanum*

In this study, 2 viewpoints have been investigated with respect to the role of (-)-catechin in the rhizosphere of *R. formosanum*. Firstly, (-)-catechin acts as antibiotic to suppress the growth of certain groups of microorganisms that are sensitive to it. Secondly, (-)-catechin increased catechin-utilizing bacteria in the rhizosphere of *R. formosanum*. Catechin, one of the major flavonoids, is widely found in natural fruit, green tea, wine, and traditional Chinese medicine [34-36]. Many plant flavonoids exhibit the functions of both antioxidants and free radicals whose role is to undertake oxygen cleaning in biological systems. They may also have antimicrobial characteristics [37-40]. Previously, the antimicrobial activity of catechin was demonstrated the concentration-dependent decline in the total culturable count numbers of the bacterial community. Catechin inhibited growth of some, but not all, of the soil bacterial population tested in the root zone of spotted knapweed [19,41]. Microorganisms are hypothesized to be most important for the productivity of nutrient poor ecosystems and a reduction in microbial diversity could reduce growth of plant species and plant diversity [42]. In this study, the population of total culturable bacteria was suppressed as the concentration of catechin increased in the soil. The dominant bacteria in the rhizosphere of *R. formosanum* were restricted to the gram-negative proteobacteria. Thus, it is concluded that catechin generated the selective pressure that drove microbial diversity.

Catechin acts not only as an antibiotic to suppress the microorganisms, but also as a carbon source for microorganisms in the rhizosphere. Catechin-utilizing bacteria can transform (-)-catechin into glycerol, a common carbon source for bacteria without passive transport system. Glycerol is one of the few compounds that can enter bacteria by facilitated diffusion without the need for energy. Therefore, microorganisms can directly utilize glycerol without any transport system [43]. The results of this study strongly suggest that catechin was the carbon source for catechin-utilizing bacteria in the rhizosphere of *R. formosanum*.

### Bacterial flora in the rhizosphere of *R. formosanum*

By culture-dependent methods, the catechin-utilizing bacteria *Pseudomonas* spp., *Burkholderia* spp. and *Variovorax* spp., were found to be abundant in the rhizosphere and were closely related to uncultured clones. In the *Pseudomonadaceae*, the similarity of 16S rRNA sequences between type strain and all the *Pseudomonas* strains we isolated in this study were less than 98% (data not shown). According to previously proposed hypothesis [44], the similarity below 98.6% provides evidence for different genospecies with a high level of confidence. Further investigations with more detail experiments including G+C content, composition of cellular fatty acids and DNA hybridization are needed for this newly *Pseudomonas* sp. identification. The ability of proteobacteria to degrade aromatic compounds, including protocatechuic acid, catechol, and hydroxybenzoic acid, has been reported previously [45]. Aromatic oxygenase-encoding genes have also been identified in the ortho ring cleavage pathway of protocatechuic acid and catechol [45,46]. The high levels of phenolic compounds in the organic layer of *R. formosanum* could be the carbon source for the β-proteobacteria found in the rhizosphere. Interestingly, fungi and the dominant genus in the rhizosphere, *Herbaspirillum*, did not utilize the catechin. Previously, fungi shown to be closely associated with *R. formosanum* were identified as ericoid mycorrhizal endophytes that were capable of degrading organic matter for growth [22]. The genus *Herbaspirillum* is composed of nitrogen-fixing bacterial species and has been found in the roots and stems of rice, sorghum, maize and sugarcane [47]. This suggests that the role of the endophytic *Herbaspirillum* species, in the nitrogen–limited environment of *R. formosanum*, would fix nitrogen for plant growth. In conclusion, (-)-catechin suppressed certain groups of microorganisms and increased catechin-utilizing bacteria in the rhizosphere of *R. formosanum*.

### The impact of microbial biotransformation of (-)-catechin

The acidic soils found in the rhizosphere of *R. formosanum* could be attributed to the accumulation of phenolic compounds from metabolites and compounds released from the decomposition of organic matter in the soil. During the catechin biotransformation process, the product formed was confirmed as protocatechuic acid, which was also found abundantly in the soil organic matter layer of *R. formosanum* [20]. Previous research on catechin degradation revealed that *Pseudomonas* sp. converted catechin into protocatechuic acid, catechol, and phloroglucinol carboxylic acid [48,49]. In *Acinetobacter calcoaceticus*, phloroglucinol carboxylic acid and protocatechuic acid appeared as intermediates in catechin degradation [50,51]. Other end products, including maleylacetate, ketoadipate, and carboxy muconate, were generated by a variety of other microorganisms, including *Aspergillus* spp., *Bradyrhizobium* spp., *Fusarium* spp., *Rhizobium* spp., and *Streptomyces* spp., during catechin degradation [52]. In this study, the results showed that catechin was converted into protocatechuic acid via the action of *Pseudomonas* sp.. Protocatechuic acid has been reported as an allelochemical [53,54] and exhibits a stronger inhibitory effect than that of (-)-catechin. A reduction in ETR_max_ of over 60% was observed when plants were exposed to protocatechuic acid. An increase in F_0_ and decrease in F_v_/F_m_ indicated the occurrence of photoinhibitory damage, and this phenomenon also been observed when plants treated with other phenolic compounds [55]. Moreover, a mixture of protocatechuic acid (50 µg/g soil) and (-)-catechin (10~1000 µg/g soil) inhibited seed germination by over 50%. Biotransformation intermediates could play an active role in allelopathic interactions. Indeed, the successful stabilization of *R. formosanum* might be due to the synergistic phytotoxic effects of protocatechuic acid or other biotransformation intermediates rather than those of the original chemicals, such as catechin. 

### Hypothesis: The allelopathic interactions between *R. formosanum* and dominant *Pseudomonas* species

**Figure 6:**
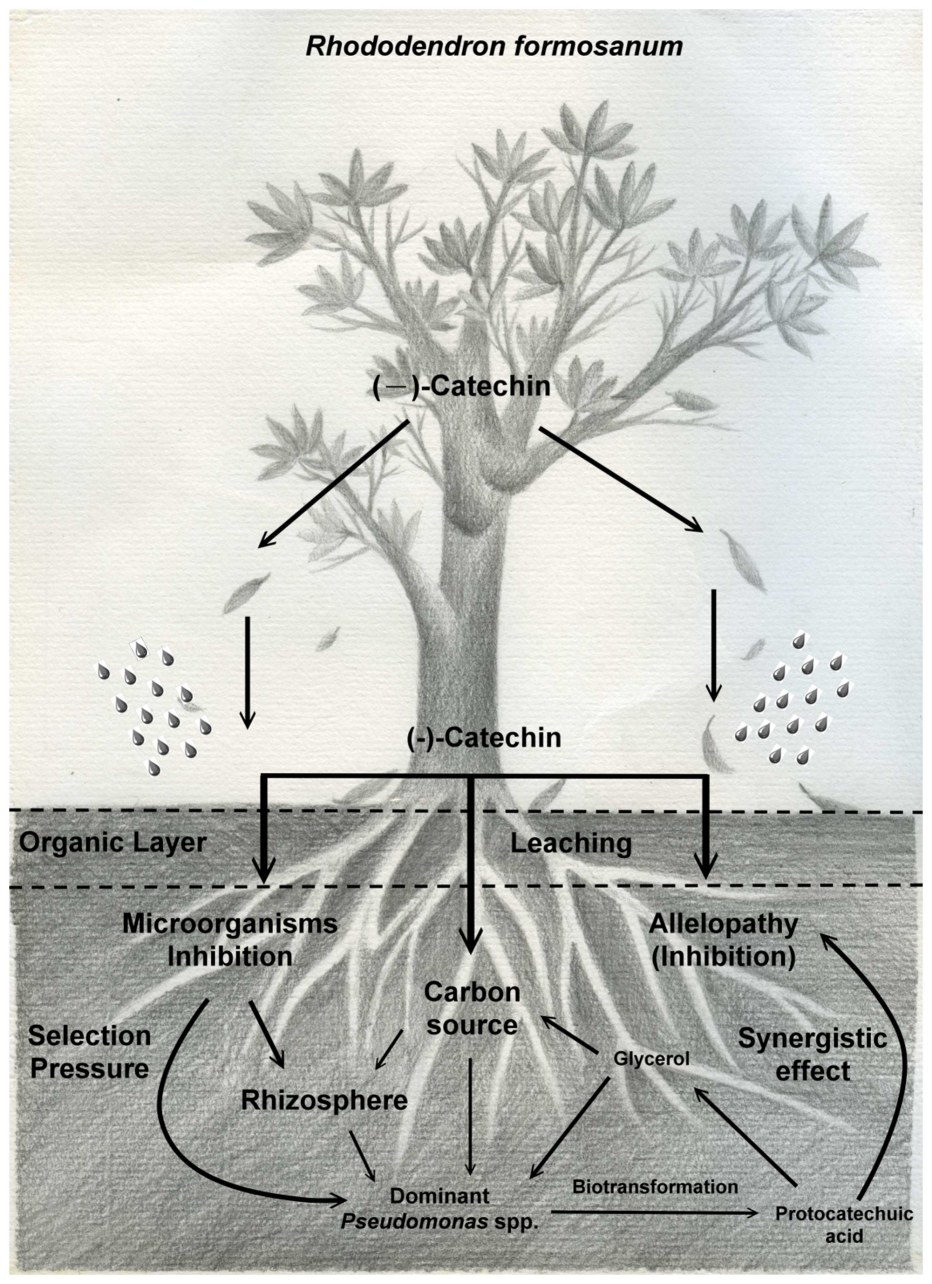
Hypothetical scheme of allelopathic interactions between *Rhododendron formosanum* and dominant *Pseudomonas* species. Initially, (-)-catechin from the leaves of *R. formosanum* accumulates in the soil via leaching. After a period of time, the population of catechin-utilizing *Pseudomonas* spp. increases and the (-)-catechin is converted into protocatechuic acid through biotransformation. The protocatechuic acid exhibited synergistic inhibitory effects with the original (-)-catechin on the seed germination of the plant. Finally, (-)-catechin is transformed into glycerol and utilized by microorganisms as a carbon source. Thus, interactions between *R. formosanum* and the dominant *Pseudomonas* species are established.

A complete understanding of the allelopathic phenomenon requires the study of degradation or biotransformation products of allelochemicals. When allelochemicals come into contact with different microorganisms, they can get transformed to new products. Soil organisms influence allelopathic effects, especially when the release of allelochemicals is brought about by decomposition of plant residues. Previous studies indicated plant litter decomposition is a key process in terrestrial ecosystems, including carbon turnover and nutrient releasing [56]. Soil microorganisms affect aboveground litter decomposition and represent a biotic component affecting nutrient releasing [57]. In this study, the below-ground interactions were also discussed. Overall, as shown in Figure 6, we have summarized the presence of allelopathic interactions between *R. formosanum* and dominant *Pseudomonas* species in this study. Initially, (-)-catechin from the leaves of *R. formosanum* accumulates in the soil via strong leaching and inhibits the growth of understory plants and microorganisms in the soil. After a period of time, the population of catechin-utilizing microorganisms increases and the (-)-catechin is converted into different intermediates such as protocatechuic acid through biotransformation. The protocatechuic acid exhibits stronger phytotoxic effects than the original (-)-catechin on seed germination, root length, and PSII of plants. Protocatechuic acid also exhibits synergistic inhibitory effects with (-)-catechin on seed germination of plants at low concentrations. Thus, a pure stand of *R. formosanum* is established. The allelopathic interaction between the plant and microorganisms was shown by the circulation of (-)-catechin between *Pseudomonas* spp. and *R. formosanum*. 

## Conclusions

Plants in their natural habitat are continuously in contact with different microorganisms. It has been demonstrated that plants produce an array of chemicals and that many of these chemicals leach into the rhizosphere and have allelopathic effects on soil conditions, neighbouring plants, and microorganisms. Microorganisms with a high resistance to these antimicrobial chemicals survive and, in some cases, the chemicals are utilized as nutrient sources, which can lead to an increase in the population of the selected microorganisms. This study showed that during decomposition, the intermediate compounds of biotransformation had synergistic allelopathic effects with the original compounds. Chemical, biological, ecological and microbiological analyses not only revealed allelopathic interactions between the plants and microorganisms, but also provided a model for understanding the successful invasion of new areas by alien plants. Further studies on the role of other microorganisms in the allelopathic effects of *R. formosanum* are needed and the identification of bacterial metabolites from those catechin-utilizing bacteria could reveal further aspects of the interactions between plants and microorganisms.

## Supporting Information

Text S1 **Allelochemicals identification.**
(DOC)

Figure S1 **The phylogenetic tree of γ-proteobacteria by 16S rRNA gene sequencing.** The cluster was constructed by neighbour-joining methodology and 1000 bootstrap analysis. The bootstrap values are shown as a percentage in the tree. Catechin-utilizing bacteria isolated from 3 sampling sites of *R. formosanum* were assigned as CRF3 and uncultured bacteria identified by direct DNA extraction from 3 sampling sites of *R. formosanum* were assigned as RF3. Genbank accession numbers of type strains were assigned following the name of the bacteria.
(JPG)

Figure S2 **The phylogenetic tree of β-proteobacteria by 16S rRNA gene sequencing.** The cluster was constructed by neighbour-joining methodology and 1000 bootstrap analysis. The bootstrap values are shown as a percentage in the tree. Catechin-utilizing bacteria isolated from 3 sampling sites of *R. formosanum* were assigned as CRF3 and uncultured bacteria identified by direct DNA extraction from 3 sampling sites of *R. formosanum* were assigned RF3. Genbank accession numbers of type strains were assigned following the name of the bacteria.
(JPG)

Figure S3 **ESI-MS/MS analysis of taxifolin.** The molecular weight of compound was identified as 304 by electrospray in negative or positive ion mode, which generated the deprotonated molecule, [M-H]^-^ (*m/z* 303), or the sodium adduct, [M+Na] (*m/z* 327). In tandem mass spectrometric mode, taxifolin produced a deprotonated ion (*m/z* 303), a fragment corresponding to deprotonated luteolin (*m/z* 285), 5,7-dihydroxychromone (*m/z* 177) and 1,3,5-trihydroxybenzene (*m/z* 125).
(JPG)

Figure S4 **ESI-MS/MS analysis of protocatechuic acid.** The molecular weight of compound was identified as 154 by electrospray in negative ion mode, which generated the deprotonated molecule [M-H]^-^ (*m/z* 153). In tandem mass spectrometric mode, loss of CO_2_ was observed for protocatechuic acid and the characteristic [M-H-44] ^-^ (*m/z* 109) ion was formed.
(JPG)

Figure S5 **ESI-MS/MS analysis of the biotransformation process for catechin.** The molecular weight of catechin was identified as 290 by electrospray in negative or positive ion mode, which generated the deprotonated molecule, [M-H]^-^ (*m/z* 289) or the sodium adduct [M+Na] (*m/z* 313). The molecular weight of the major product produced in catechin biotransformation was 92, which corresponded to the sodium adduct [M+Na] (*m/z* 115), present in glycerol.
(JPG)

Figure S6 **The ^1^H NMR spectra analysis of the biotransformation process for catechin.** Ten mg of crude enzyme from *Pseudomonas* sp. CRF3-Ps-1 was incubated with 20 mg catechin. After 24 h incubation, the enzymatic reaction was terminated and subjected to NMR analysis in D_2_O. As shown in this figure, the catechin and glycerol co-existed in the reaction. The spectra from 3.44 to 3.85 ppm were identified as glycerol signals.
(JPG)

Figure S7 **Effects of protocatechuic acid (PCA) and catechin (CAT) on photosynthetic capacity.** Changes in the chlorophyll fluorescence parameters F_v_/F_m_ (maximum quantum efficiency of PSII), F_0_ (initial fluorescence), F_m_ (maximum fluorescence), and ETR_max_ (maximum electron transport rate) after 1 week of exposure to 4 concentrations of protocatechuic acid and catechin. Every column in each graph represents the mean (± SE) values of 3 replicates. *p < 0.05, **p < 0.01, ***p < 0.005.
(JPG)

Figure S8 **Dynamics of (-)-catechin degraded by way of soil incubation.** Standard (-)-catechin was added in soil at known concentrations (200 µg/g soil). Relative concentrations of (-)-catechin was measured during 120 h incubation with fresh soil from the rhizosphere of *R. formosanum*.
(JPG)

Table S1 **^1^H NMR and ^13^C NMR data (δ, ppm) of (-)-catechin in CD_3_OD compared with literature.**
(DOC)

Table S2 **^1^H NMR and ^13^C NMR data (δ, ppm) of taxifolin in CD_3_OD compared with literature.**
(DOC)

Table S3 **^1^H NMR and ^13^C NMR data (δ, ppm) of protocatechuic acid in CD_3_OD compared with literature.**
(DOC)

Table S4 **^1^H NMR and ^13^C NMR data (δ, ppm) of glycerol in CD_3_OD compared with literature.**
(DOC)

Table S5 **Liquid chromatography-electrospray ionization /tandem mass spectrometry (LC-ESI-MS/MS) characteristics of (-)-catechin, biotransformation intermediates and their corresponding metabolites.**
(DOC)

Table S6 **The synergistic effects of (-)-catechin (CAT) and protocatechuic acid (PCA) on the seed germination and radicle growth of *L. sativa* at different combinations.** Error bars are ± SE of the mean. Post Duncan’s multiple range test was also used to evaluate the means; those, with the same letters are not significantly different at the α = 0.05 level.
(DOC)
