# Supplementary material for: The Impact of Microbial Biotransformation of Catechin in Enhancing the Allelopathic Effects of Rhododendron formosanum
Source: PLoS One. 2013 Dec 31;8(12):e85162. doi: 10.1371/journal.pone.0085162 (PMC3877349; doi:10.1371/journal.pone.0085162)
Supplement: Text S1 — Allelochemicals identification. (DOC) [file pone.0085162.s001.doc]

**Text S1.** Allelochemicals identification

In order to understand the biotransformation of (-)-catechin, the dominant *Pseudomonas* sp. CRF3-Ps-1 was selected as a model bacterium to investigate the effects of allelochemicals, including biotransformation intermediates and bacterial metabolites. Several catechin biotransformation intermediates were isolated and identified by NMR spectroscopy and LC-ESI- MS/MS (Tables S1-S5; Figs S3-S6). On LC-ESI-MS/MS fragment analysis (Tables S1-S5; Figs S3-S6), the spectra generated by the compounds in this study gave the deprotonated molecule [M-H]^－^, the protonated molecule [M+H]^＋^, and the sodium adduct [M+Na]^＋^(Table S5). The molecular ions were then subjected to full-scan MS analysis over an *m/z* range of 50–1000. In tandem mass spectrometric mode, catechin produced the deprotonated form [M-H]^－^ (*m/z* 289) and lost a CH_2_CHOH (*m/z* 245) group, which confirmed the results reported by Sanchez-Rabaneda *et al*. (2003). Taxifolin contained the deprotonated ion (*m/z* 303), and fragments corresponding to deprotonated luteolin (*m/z* 285), 5,7-dihydroxychromone (*m/z* 177), and 1,3,5-trihydroxybenzene (*m/z* 125) . CO_2_ loss was observed in protocatechuic acid and, as a result, the characteristic [M-H-44]^－^ (*m/z* 109) ion was formed. . ESI ionization was more sensitive in the positive mode than in the negative mode for glycerol ionization. For example, a [M+Na]^＋^ (*m/z* 115) ion was generated as a characteristic ion for glycerol in the positive mode.

1. Sawada Y, Akiyama K, Sakata A, Kuwahara A, Otsuki H, et al. (2009) Widely targeted metabolomics based on large-scale MS/MS data for elucidating metabolite accumulation patterns in plants. Plant Cell Physiol 50: 37-47.

2. Sanchez-Rabaneda F, Jauregui O, Casals I, Andres-Lacueva C, Izquierdo-Pulido M, et al. (2003) Liquid chromatographic/electrospray ionization tandem mass spectrometric study of the phenolic composition of cocoa (Theobroma cacao). J Mass Spectrom 38: 35-42.
