# Supplementary figures and images for: The Impact of Microbial Biotransformation of Catechin in Enhancing the Allelopathic Effects of Rhododendron formosanum
Source: PLoS One. 2013 Dec 31;8(12):e85162. doi: 10.1371/journal.pone.0085162 (PMC3877349; doi:10.1371/journal.pone.0085162)

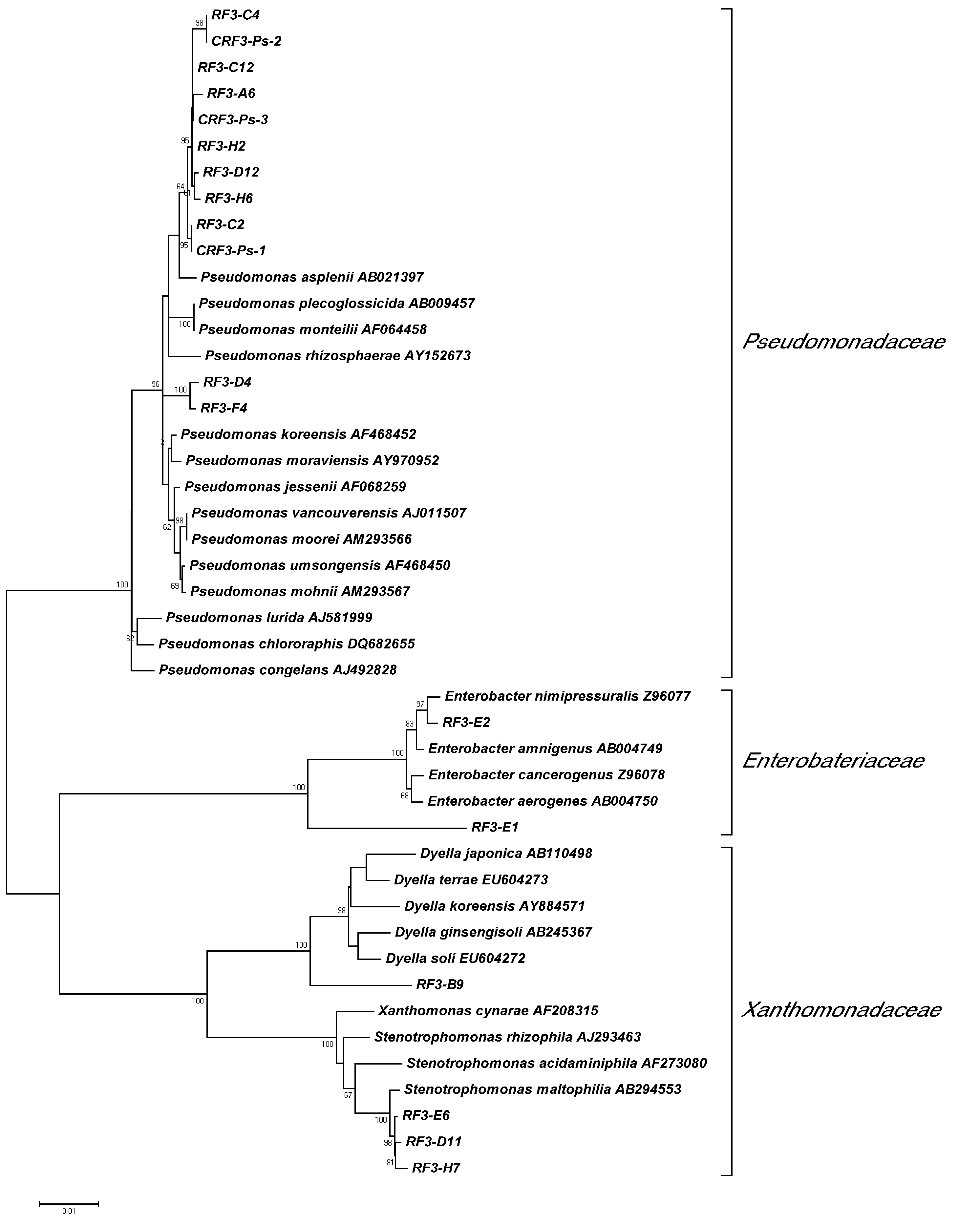

Supplement: Figure S1 — The phylogenetic tree of γ-proteobacteria by 16S rRNA gene sequencing. The cluster was constructed by neighbour-joining methodology and 1000 bootstrap analysis. The bootstrap values are shown as a percentage in the tree. Catechin-utilizing bacteria isolated from 3 sampling sites of R. formosanum were assigned as CRF3 and uncultured bacteria identified by direct DNA extraction from 3 sampling sites of R. formosanum were assigned as RF3. Genbank accession numbers of type strains were assigned following the name of the bacteria. (JPG) [file pone.0085162.s002.jpg]

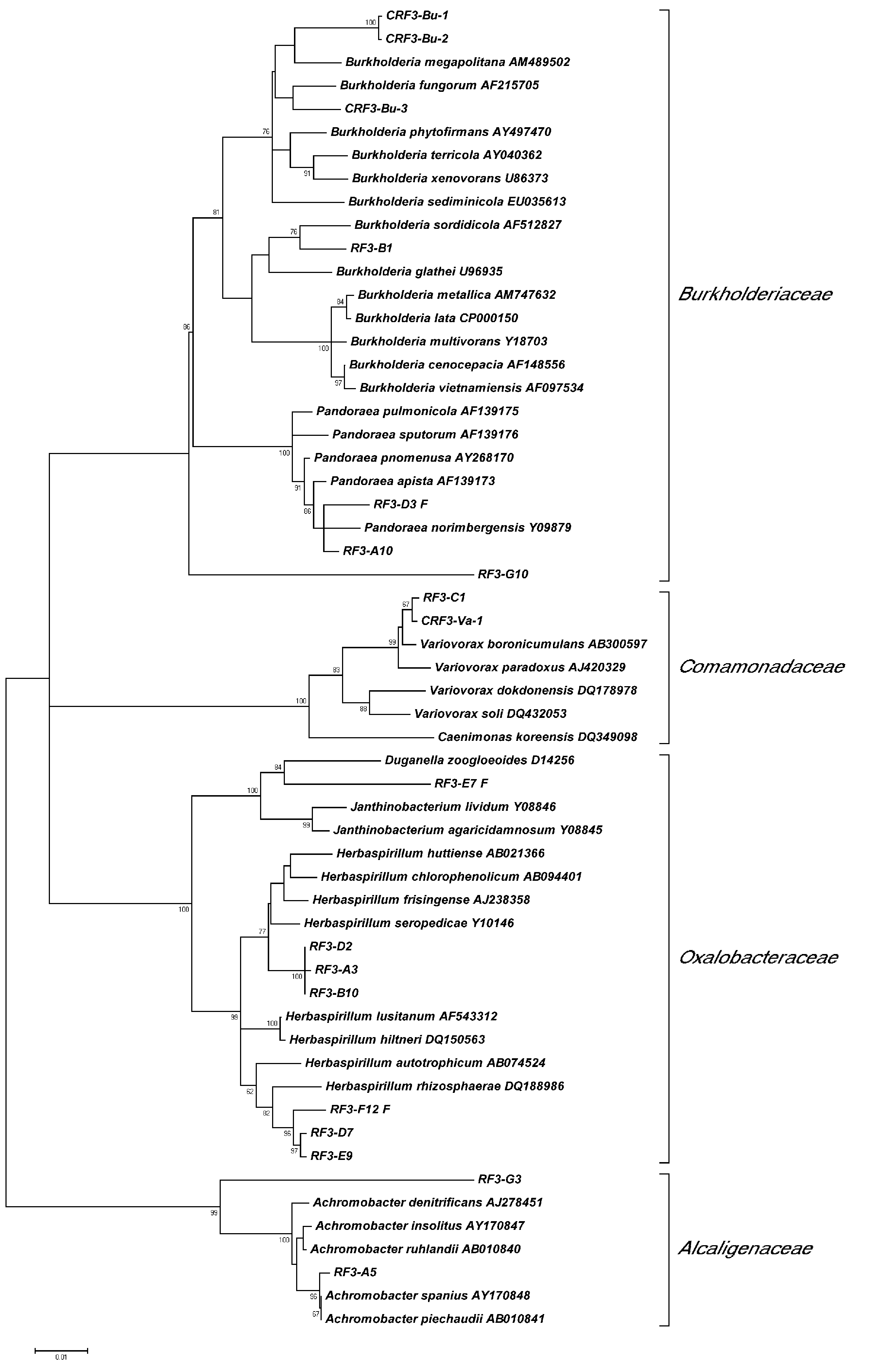

Supplement: Figure S2 — The phylogenetic tree of β-proteobacteria by 16S rRNA gene sequencing. The cluster was constructed by neighbour-joining methodology and 1000 bootstrap analysis. The bootstrap values are shown as a percentage in the tree. Catechin-utilizing bacteria isolated from 3 sampling sites of R. formosanum were assigned as CRF3 and uncultured bacteria identified by direct DNA extraction from 3 sampling sites of R. formosanum were assigned RF3. Genbank accession numbers of type strains were assigned following the name of the bacteria. (JPG) [file pone.0085162.s003.jpg]

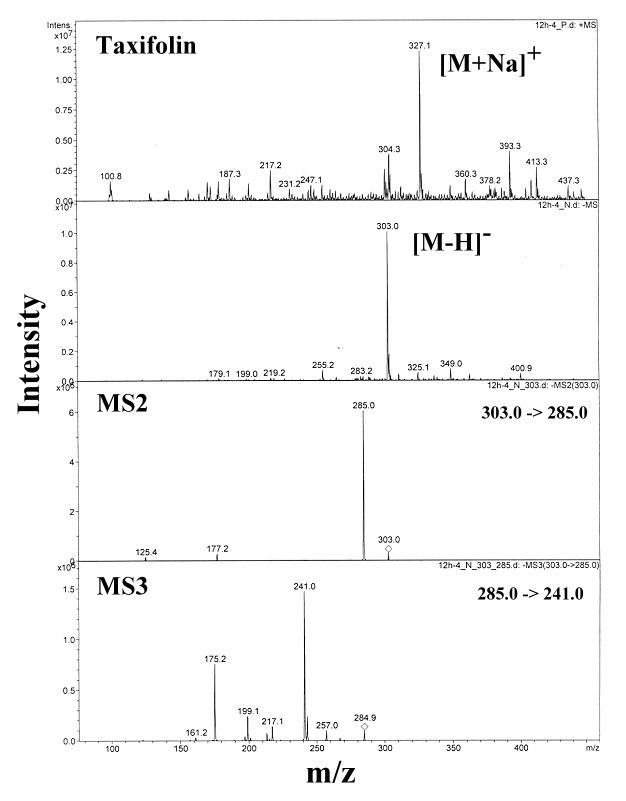

Supplement: Figure S3 — ESI-MS/MS analysis of taxifolin. The molecular weight of compound was identified as 304 by electrospray in negative or positive ion mode, which generated the deprotonated molecule, [M-H]- (m/z 303), or the sodium adduct, [M+Na] (m/z 327). In tandem mass spectrometric mode, taxifolin produced a deprotonated ion (m/z 303), a fragment corresponding to deprotonated luteolin (m/z 285), 5,7-dihydroxychromone (m/z 177) and 1,3,5-trihydroxybenzene (m/z 125). (JPG) [file pone.0085162.s004.jpg]

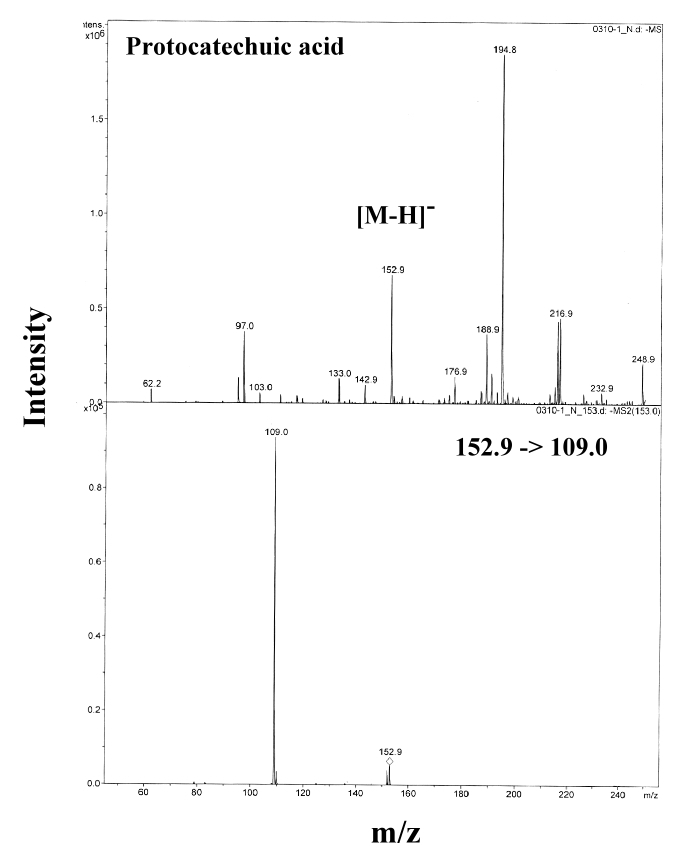

Supplement: Figure S4 — ESI-MS/MS analysis of protocatechuic acid. The molecular weight of compound was identified as 154 by electrospray in negative ion mode, which generated the deprotonated molecule [M-H]- (m/z 153). In tandem mass spectrometric mode, loss of CO2 was observed for protocatechuic acid and the characteristic [M-H-44] - (m/z 109) ion was formed. (JPG) [file pone.0085162.s005.jpg]

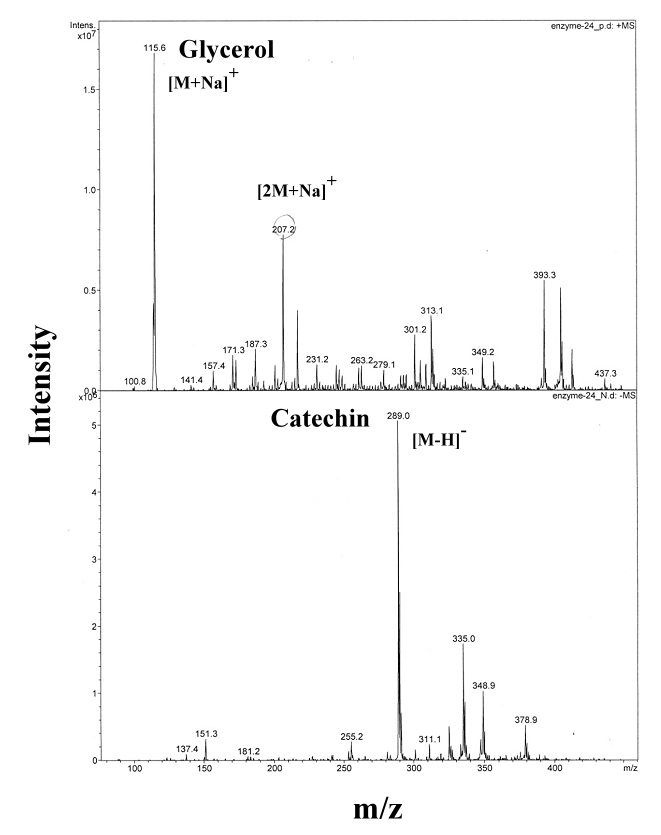

Supplement: Figure S5 — ESI-MS/MS analysis of the biotransformation process for catechin. The molecular weight of catechin was identified as 290 by electrospray in negative or positive ion mode, which generated the deprotonated molecule, [M-H]- (m/z 289) or the sodium adduct [M+Na] (m/z 313). The molecular weight of the major product produced in catechin biotransformation was 92, which corresponded to the sodium adduct [M+Na] (m/z 115), present in glycerol. (JPG) [file pone.0085162.s006.jpg]

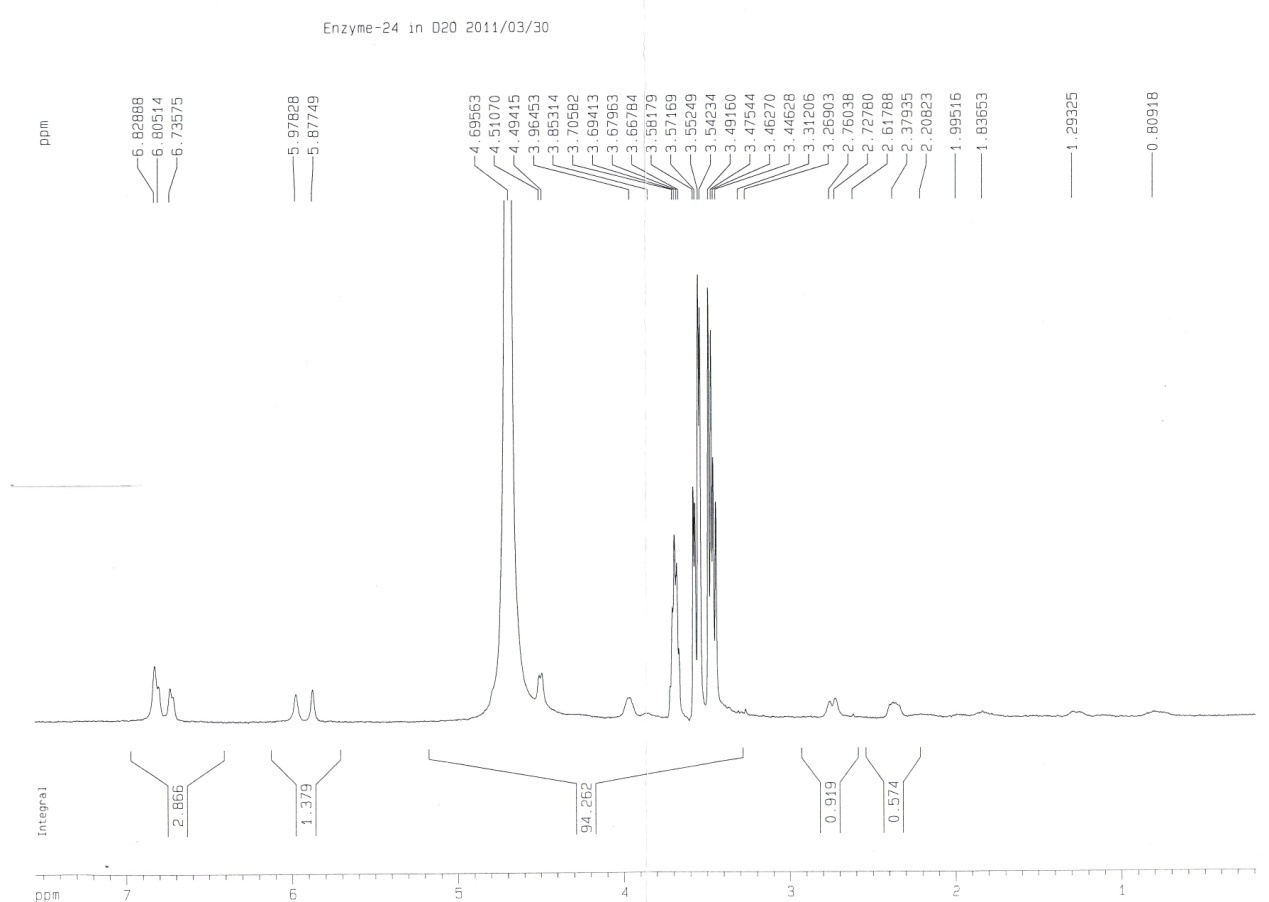

Supplement: Figure S6 — The 1H NMR spectra analysis of the biotransformation process for catechin. Ten mg of crude enzyme from Pseudomonas sp. CRF3-Ps-1 was incubated with 20 mg catechin. After 24 h incubation, the enzymatic reaction was terminated and subjected to NMR analysis in D2O. As shown in this figure, the catechin and glycerol co-existed in the reaction. The spectra from 3.44 to 3.85 ppm were identified as glycerol signals. (JPG) [file pone.0085162.s007.jpg]

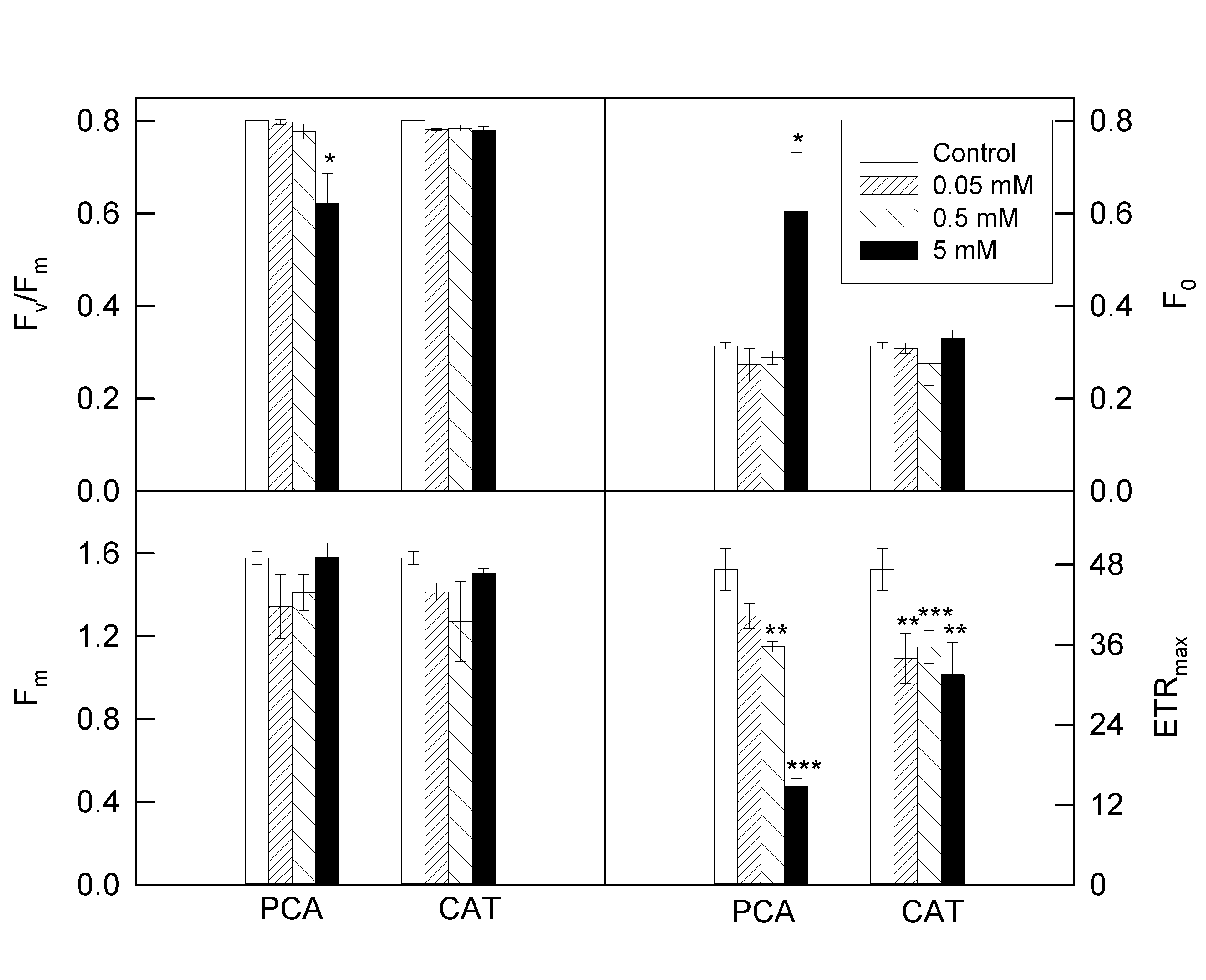

Supplement: Figure S7 — Effects of protocatechuic acid (PCA) and catechin (CAT) on photosynthetic capacity. Changes in the chlorophyll fluorescence parameters Fv/Fm (maximum quantum efficiency of PSII), F0 (initial fluorescence), Fm (maximum fluorescence), and ETRmax (maximum electron transport rate) after 1 week of exposure to 4 concentrations of protocatechuic acid and catechin. Every column in each graph represents the mean (± SE) values of 3 replicates. *p < 0.05, **p < 0.01, ***p < 0.005. (JPG) [file pone.0085162.s008.jpg]

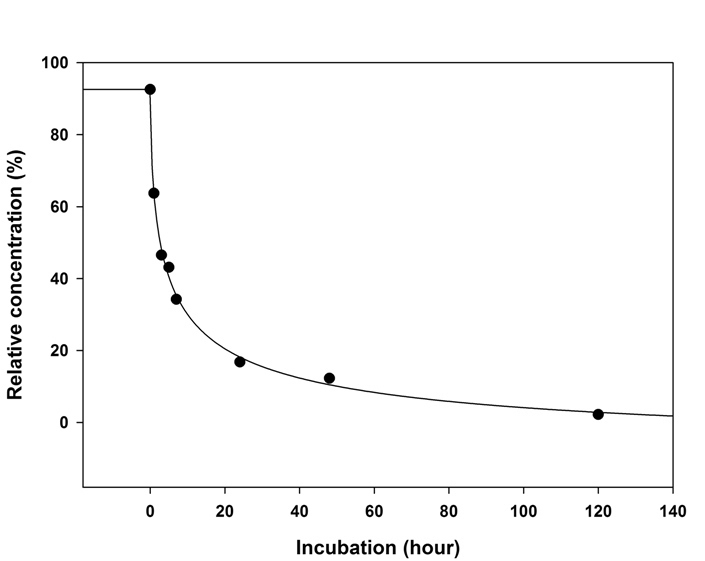

Supplement: Figure S8 — Dynamics of (-)-catechin degraded by way of soil incubation. Standard (-)-catechin was added in soil at known concentrations (200 µg/g soil). Relative concentrations of (-)-catechin was measured during 120 h incubation with fresh soil from the rhizosphere of R. formosanum. (JPG) [file pone.0085162.s009.jpg]
