# Supplementary material for: The Impact of Microbial Biotransformation of Catechin in Enhancing the Allelopathic Effects of Rhododendron formosanum
Source: PLoS One. 2013 Dec 31;8(12):e85162. doi: 10.1371/journal.pone.0085162 (PMC3877349; doi:10.1371/journal.pone.0085162)
Supplement: Table S1 — 1H NMR and 13C NMR data (δ, ppm) of (-)-catechin in CD3OD compared with literature. (DOC) [file pone.0085162.s010.doc]

**Table S1.** ^1^H NMR and ^13^C NMR data (δ, ppm) of (-)-catechin in CD_3_OD compared with literature

| Position | ^1^H, ppm (Hz) | ^1^H (literature) | ^13^C | ^13^C (literature) |
| --- | --- | --- | --- | --- |
| 2 | 4.55(dd, *J*= 7.5) | 4.6(dd, *J*= 7.2) | 82.8(d) | 82.5(d) |
| 3 | 3.96(m) | 4.0(m) | 68.8(d) | 68.6(d) |
| 4 | 2.49(dd, *J* = 8.1, 16.1) 2.83(dd, *J* = 5.4, 16.1) | 2.53(dd, *J* = 8, 16) 2.86(dd, *J* = 5.6, 16) | 28.5(t) | 28.1(t) |
| 4a |  |  | 100.8(s) | 100.8(s) |
| 5 |  |  | 157.5(s) | 157.3(s) |
| 6 | 5.91(d, *J* = 2.2) | 5.97(d, *J* = 2.4) | 96.3(d) | 96.3(d) |
| 7 |  |  | 157.7(s) | 157.4(d) |
| 8 | 5.84(d, *J* = 2.2) | 5.90(d, *J* = 2.4) | 95.5(d) | 95.5(d) |
| 8a |  |  | 156.8(s) | 156.7(s) |
| 1’ |  |  | 132.1(s) | 132.0(s) |
| 2’ | 6.83(d, *J* = 1.6) | 6.86(d, *J* = 2) | 115.2(d) | 115.1(d) |
| 3’ |  |  | 146.2(s) | 145.9(s) |
| 4’ |  |  | 146.2(s) | 145.9(s) |
| 5’ | 6.76(d, *J* = 8.1) | 6.78(d, *J* = 8) | 116.0(d) | 116.1(d) |
| 6’ | 6.70(dd, *J* = 1.8, 8.1) | 6.72(dd, *J* = 2, 8) | 120.0(d) | 120.0(d) |
