# Supplementary material for: The Impact of Microbial Biotransformation of Catechin in Enhancing the Allelopathic Effects of Rhododendron formosanum
Source: PLoS One. 2013 Dec 31;8(12):e85162. doi: 10.1371/journal.pone.0085162 (PMC3877349; doi:10.1371/journal.pone.0085162)
Supplement: Table S2 — 1H NMR and 13C NMR data (δ, ppm) of taxifolin in CD3OD compared with literature. (DOC) [file pone.0085162.s011.doc]

**Table S2.** ^1^H NMR and ^13^C NMR data (δ, ppm) of taxifolin in CD_3_OD compared with literature

| Position | ^1^H, ppm (Hz) | ^1^H (literature) | ^13^C | ^13^C (literature) |
| --- | --- | --- | --- | --- |
| 2 | 4.90(d, *J*= 11.5) | 4.90(d, *J*= 11) | 85.1(d) | 83.9(d) |
| 3 | 4.49(d, *J* = 11.5) | 4.50(d, *J* = 11) | 73.7(d) | 72.5(d) |
| 4 |  |  | 198.4(t) | 197.6(t) |
| 4a |  |  | 101.8(s) | 101.0(s) |
| 5 |  |  | 165.3(s) | 164.3(s) |
| 6 | 5.87(d, *J* = 1.8) | 5.88(d, *J* = 1.9) | 97.3(d) | 96.4(d) |
| 7 |  |  | 168.8(s) | 167.2(s) |
| 8 | 5.91(d, *J* = 1.9) | 5.92(d, *J* = 2.0) | 96.3(d) | 95.4(d) |
| 8a |  |  | 164.5(s) | 163.5(s) |
| 1’ |  |  | 129.9(s) | 129.1(s) |
| 2’ | 6.95(d, *J* = 1.5) | 6.97(d, *J* = 1.6) | 115.9(d) | 115.1(d) |
| 3’ |  |  | 146.3(s) | 145.1(s) |
| 4’ |  |  | 147.1(s) | 145.9(s) |
| 5’ | 6.81(d, *J* = ) | 6.80(dd, *J* = 4.6, 4.6) | 116.1(d) | 115.2(d) |
| 6’ | 6.83(dd, *J* = 1.6, 1.6) | 6.88(dd, *J* = 1.6, 1.6) | 120.9(d) | 120.2(d) |
