# Supplementary material for: The Impact of Microbial Biotransformation of Catechin in Enhancing the Allelopathic Effects of Rhododendron formosanum
Source: PLoS One. 2013 Dec 31;8(12):e85162. doi: 10.1371/journal.pone.0085162 (PMC3877349; doi:10.1371/journal.pone.0085162)
Supplement: Table S3 — 1H NMR and 13C NMR data (δ, ppm) of protocatechuic acid in CD3OD compared with literature. (DOC) [file pone.0085162.s012.doc]

**Table S3.** ^1^H NMR and ^13^C NMR data (δ, ppm) of protocatechuic acid in CD_3_OD compared with literature

| Position | ^1^H, ppm (Hz) | ^1^H (literature) | ^13^C | ^13^C (literature) |
| --- | --- | --- | --- | --- |
| 1 |  |  | 123.0 | 121.3 |
| 2 | 7.44(d, *J* =1.9) | 7.48 (d, *J* =2.0) | 117.7 | 116.6 |
| 3 |  |  | 145.9 | 144.9 |
| 4 |  |  | 151.5 | 150.1 |
| 5 | 7.41(dd, *J* =8.3, 1.9) | 7.42 (dd, *J* =8.2, 2.0) | 115.8 | 115.8 |
| 6 | 6.79(d, *J* =8.0) | 6.82 (d, *J* =8.2) | 123.9 | 122.4 |
| 7 |  |  | 170.3 | 167.7 |
