# Supplementary material for: The Impact of Microbial Biotransformation of Catechin in Enhancing the Allelopathic Effects of Rhododendron formosanum
Source: PLoS One. 2013 Dec 31;8(12):e85162. doi: 10.1371/journal.pone.0085162 (PMC3877349; doi:10.1371/journal.pone.0085162)
Supplement: Table S4 — 1H NMR and 13C NMR data (δ, ppm) of glycerol in CD3OD compared with literature. (DOC) [file pone.0085162.s013.doc]

**Table S4.** ^1^H NMR and ^13^C NMR data (δ, ppm) of glycerol in CD_3_OD compared with literature

| Position | ^1^H, ppm (Hz) | ^1^H (literature) | ^13^C | ^13^C (literature) |
| --- | --- | --- | --- | --- |
| 1 | 3.58 (dd, J=11.2, 6.04) | 3.54 (dd, J=11.2, 6.10) | 64.34 | 64.79 |
| 2 | 3.64 (dd, J=11.2, 4.78) | 3.61 (dd, J=11.2, 4.90) | 73.80 | 74.24 |
| 3 | 3.58 (dd, J=11.2, 6.04) | 3.54 (dd, J=11.2, 6.10) | 64.34 | 64.79 |
