# Supplementary material for: The Impact of Microbial Biotransformation of Catechin in Enhancing the Allelopathic Effects of Rhododendron formosanum
Source: PLoS One. 2013 Dec 31;8(12):e85162. doi: 10.1371/journal.pone.0085162 (PMC3877349; doi:10.1371/journal.pone.0085162)
Supplement: Table S5 — Liquid chromatography-electrospray ionization /tandem mass spectrometry (LC-ESI-MS/MS) characteristics of (-)-catechin, biotransformation intermediates and their corresponding metabolites. (DOC) [file pone.0085162.s014.doc]

**Table S5.** Liquid chromatography-electrospray ionization /tandem mass spectrometry (LC-ESI-MS/MS) characteristics of (-)-catechin, biotransformation intermediates and their corresponding metabolites

| Compounds | *Rt* (min) | M. W. | Detecting ion | Fragments |
| --- | --- | --- | --- | --- |
| Glycerol | 1.7 | 92 | [M+Na]^+^: 115 | - |
| Protocatechuic acid | 7.8 | 154 | [M-H]^-^: 152.6 | 108.7 |
| Catechin | 10.8 | 290 | [M-H]^-^: 288.8 | 224.7, 204.6 |
| Taxifolin | 22.4 | 304 | [M-H]^-^: 302.8 | 285.0, 177.2, 125.4 |
