# Supplementary material for: The Impact of Microbial Biotransformation of Catechin in Enhancing the Allelopathic Effects of Rhododendron formosanum
Source: PLoS One. 2013 Dec 31;8(12):e85162. doi: 10.1371/journal.pone.0085162 (PMC3877349; doi:10.1371/journal.pone.0085162)
Supplement: Table S6 — The synergistic effects of (-)-catechin (CAT) and protocatechuic acid (PCA) on the seed germination and radicle growth of L. sativa at different combinations. Error bars are ± SE of the mean. Post Duncan’s multiple range test was also used to evaluate the means; those, with the same letters are not significantly different at the α = 0.05 level. (DOC) [file pone.0085162.s015.doc]

**Table S6.** The synergistic effects of (-)-catechin (CAT) and protocatechuic acid (PCA) on the seed germination and radicle growth of *L. sativa* at different combinations. Error bars are ± SE of the mean. Post Duncan’s multiple range test was also used to evaluate the means; those, with the same letters are not significantly different at the α = 0.05 level.

|  | CAT  (μg/g soil) | PCA  (μg/g soil) | Germination (%) | Inhibition (%)* | Root length (mm) |
| --- | --- | --- | --- | --- | --- |
| Treatment 1 | 0 | 0 | 100 ± 0.0 a | (0) | 28.0 ± 1.8 a |
|  | 10 | 0 | 100 ± 0.0 a | (0) | 24.9 ± 1.1 ab |
|  | 250 | 0 | 93.3 ± 3.3 ab | (6.7) | 19.2 ± 1.5 bc |
|  | 750 | 0 | 86.6 ± 3.3 ab | (13.4) | 17.6 ± 2.1 c |
| Treatment 2 | 0 | 10 | 86.6 ± 3.3 ab | (13.4) | 28.8 ± 1.2 a |
|  | 10 | 10 | 63.3 ± 3.3 cd | (36.7) | 28.0 ± 1.7 a |
|  | 250 | 10 | 63.3 ± 3.3 cd | (36.7) | 22.3 ± 1.9 abc |
|  | 750 | 10 | 46.6 ± 3.3 def | (53.4) | 19.4 ± 2.7 bc |
| Treatment 3 | 0 | 50 | 76.6 ± 3.3 bc | (23.4) | 22.2 ± 1.8 abc |
|  | 10 | 50 | 53.3 ± 8.8 de | (46.7) | 22.3 ± 4.8 abc |
|  | 250 | 50 | 36.6 ± 12.0 ef | (63.4) | 18.5 ± 2.5 bc |
|  | 750 | 50 | 30.0 ± 11.5 f | (70) | 9.6 ± 1.5 d |

*% inhibition of germination was obtained from the data for each treatment against treatment 1 with 0 μg/g soil as the control.
